# Efficient Generation of Chemically Induced Mesenchymal Stem Cells from Human Dermal Fibroblasts

**DOI:** 10.1038/srep44534

**Published:** 2017-03-17

**Authors:** Pei-Lun Lai, Hsuan Lin, Shang-Fu Chen, Shang-Chih Yang, Kuo-Hsuan Hung, Ching-Fang Chang, Hsiang-Yi Chang, Frank Leigh Lu, Yi-Hsuan Lee, Yu-Chuan Liu, Hsiao-Chun Huang, Jean Lu

**Affiliations:** 1 Genome and Systems Biology Degree Program, College of Life Science, National Taiwan University, Taipei, Taiwan; 2 Genomics Research Center, Academia Sinica, Taipei, Taiwan; 3 Department of Pediatrics, National Taiwan University Hospital and National Taiwan University Medical College, Taipei, Taiwan; 4 Institute of Molecular and Cellular Biology and Department of Life Science, College of Life Science, National Taiwan University, Taipei, Taiwan; 5 Graduate Institute of Electronics Engineering, College of Electrical Engineering and Computer Science, National Taiwan University, Taipei, Taiwan; 6 National RNAi Platform/National Core Facility Program for Biotechnology, Taipei, Taiwan; 7 Department of Life Science, Tzu Chi University, Hualien, Taiwan

## Abstract

Human mesenchymal stromal/stem cells (MSCs) are multipotent and currently undergoing hundreds of clinical trials for disease treatments. To date, no studies have generated induced MSCs from skin fibroblasts with chemicals or growth factors. Here, we established the first chemical method to convert primary human dermal fibroblasts into multipotent, induced MSC-like cells (iMSCs). The conversion method uses a defined cocktail of small molecules and growth factors, and it can achieve efficient conversion with an average rate of 38% in 6 days. The iMSCs have much higher clonogenicity than fibroblasts, and they can be maintained and expanded in regular MSC medium for at least 8 passages and further differentiated into osteoblasts, adipocytes, and chondrocytes. Moreover, the iMSCs can suppress LPS-mediated acute lung injury as effectively as bone marrow-derived mesenchymal stem cells. This finding may greatly benefit stem cell biology, cell therapy, and regenerative medicine.

Mesenchymal stromal/stem cells (MSCs) were first isolated from bone marrow and able to differentiate into multiple lineages, including bone, fat, cartilage, and fibroblasts^1^,^2^. In addition to their multipotency, MSCs are known for their immunoregulatory functions^3^ and the ability to secrete multiple cytokines to promote tissue healing^4^. Currently, hundreds of clinical trials are performed to test the efficacy of MSCs in cell therapy (www.clinicaltrials.gov).

According to the Guidelines of the Mesenchymal and Tissue Stem Cell Committee of the International Society for Cellular Therapy (ISCT), MSCs should fulfill three criteria, including (1) being plastic-adherent when maintained in standard culture conditions, (2) expressing CD105, CD73 and CD90 surface markers, and not expressing CD45, CD34, CD14/CD11b, CD79a/CD19 and HLA-DR surface molecules, and (3) able to differentiate into osteoblasts, adipocytes, and chondrocytes *in vitro*^5^. Besides, a common property of MSCs is their capacity to suppress the immune responses stimulated by endotoxin-like lipopolysaccharides (LPS)^6^,^7^. It is believed that MSCs exert their immunoregulatory effects by their paracrine ability to reduce inflammation and inhibit immune responses^6^,^8^.

Unlike many other functional cell types, due to the lack of identified tissue-specific master regulators/transcription factors in MSCs, it is hard to pinpoint a combination of key transcription factors to generate induced MSC-like cells (iMSCs) from fibroblasts. Previous studies have indicated that SSEA-4 and PODXL are two promising markers to isolate MSCs with stronger multipotency or clonogenicity^9^,^10^,^11^. Importantly, multipotency and clonogenicity are critical properties that clearly distinct MSCs from fibroblasts^12^. SSEA-4^high^ bone marrow-derived mesenchymal stem cells (BMMSCs) exhibit stronger multipotency that can differentiate into osteoblasts, chondrocytes, and adipocytes^9^,^10^. PODXL^high^ BMMSCs have higher differentiation ability and proliferative ability than PODXL^low^ BMMSCs^11^.

Chemical conversion of fibroblasts into other functional cell types has drawn substantial attention recently^13^,^14^,^15^,^16^. In this work, we report the first method to efficiently generate inducible MSC-like cells, iMSCs, from human primary dermal fibroblasts using six chemical inhibitors (6 C, including SP600125, SB202190, Go6983, Y-27632, PD0325901, and CHIR99021) with or without 3 growth factors (3GF, including transforming growth factor-β (TGF-β), basic fibroblast growth factor (bFGF), and leukemia inhibitory factor (LIF)). The chemical cocktail directly converts human fibroblasts to iMSCs with a monolayer culture in 6 days, and the conversion rate was approximately 38%. These iMSCs fulfill all criteria of MSCs defined by the ISCT^5^, and they behave like primary BMMSCs in terms of their multipotency, clonogenicity, molecular signatures, surface marker expression profile, and antisepsis function in mice. The iMSCs are multipotent and significantly decrease the fatality of endotoxin-induced acute lung injury (ALI) in a mouse model as effectively as BMMSCs. Because the conversion method does not involve any processes that may lead to insertional mutagenesis, and MSCs have a low risk of tumorigenesis, the iMSCs described here have lower safety concerns for disease treatments.

## Results

### Generation of MSC-like cells from human dermal fibroblasts using a chemical and growth factor cocktail

**Figure 1:**
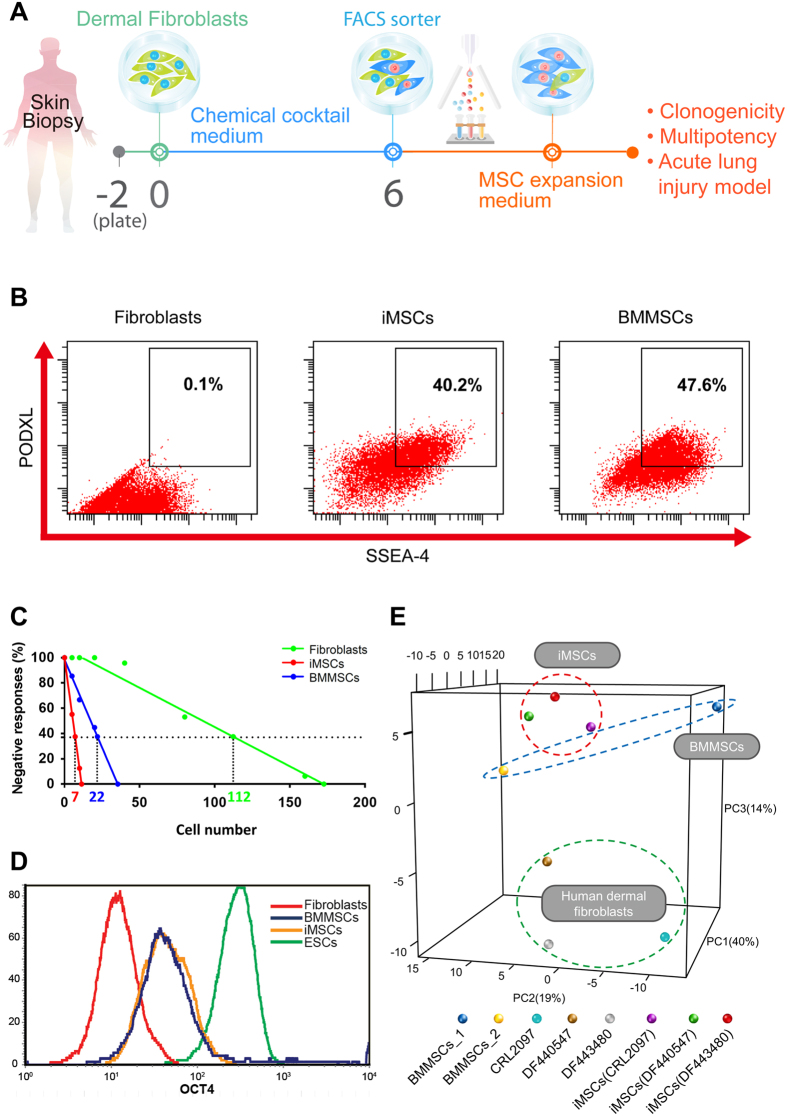
Derivation of iMSCs and the comparison of molecular signatures. (**A**) The experimental scheme for efficient derivation of iMSCs from dermal fibroblasts with 6 chemicals with or without LIF, TGF-β, and bFGF. Expanded iMSCs with clonal expansion ability (clonogenicity) can be further differentiated into different lineages (multipotency) or treat sepsis-induced acute lung injury in the mouse model. (**B**) The representative flow cytometry analysis of human dermal fibroblasts CRL2097, iMSCs, and BMMSCs with MSC functional markers SSEA-4 and PODXL. SSEA-4 and PODXL were abundantly expressed in iMSCs and BMMSCs, but not in fibroblasts. (**C**) Clonogenicity of fibroblasts, iMSCs, and BMMSCs. By a 96-well colony formation assay, quantitation of CFU-F in fibroblasts (1 per 112 cells), iMSCs (1 per 7 cells), and BMMSCs (1 per 22 cells) was obtained by the negative linear relationship between the number of seeded cells and the percentage of wells with no colonies. (**D**) Flow cytometry analysis of OCT4 protein expression in human fibroblasts, iMSCs, BMMSCs, and human embryonic stem cells (hESCs). The pluripotent marker OCT4 was at a basal level in fibroblasts (CRL2097), slightly up-regulated in iMSCs and BMMSCs, and highly expressed in hESCs. (**E**) The principal component analysis of the expression of stemness genes in three different dermal fibroblasts (CRL2097, DF440547, and DF443480), iMSCs induced from the three different fibroblasts, and two independent sources of BMMSCs (BMMSC_1: primary human bone marrow MSCs used throughout this study; BMMSC_2: publicly available gene expression data for human BMMSCs with accession number GSM1533333). Expression probes were functionally annotated with Gene Ontology (GO) and selected by text-mining for the term “stem cell maintenance” in R (418 probes). Principal component 1 accounts for 40%, principal component 2 accounts for 19%, and principal component 3 accounts for 14% of the variation in the dataset. The clustering of iMSCs derived from three different fibroblast sources between two different BMMSCs suggests the robust efficacy of the cocktail.

We used human primary dermal fibroblasts that have been passaged for less than 10 times in all our experiments. The schematic diagram illustrates the process (Fig. 1A). In the initial screen, we tested several different combinations with a panel of 30 candidate chemicals and growth factors selected from previous studies of cell conversion (Supplementary Table S1)^17^,^18^,^19^. SSEA-4 and PODXL were used as markers to isolate the potentially induced MSC-like cells with superior multipotency and expansion ability than the parental fibroblasts^9^,^10^,^11^. A cocktail containing six chemical inhibitors and three growth factors (6C+3GF) that includes SP600125 (JNK inhibitor), SB202190 (p38 inhibitor), Go6983 (protein kinase C inhibitor), Y-27632 (ROCK inhibitor), PD0325901 (ERK1/2 inhibitor), CHIR99021 (GSK3β inhibitor), transforming growth factor-β (TGF-β), basic fibroblast growth factor (bFGF), and leukemia inhibitory factor (LIF) (6C+3GF) successfully generated SSEA-4^high^PODXL^high^ MSC-like cells (Fig. 1B). An important characteristic of MSCs compared to fibroblasts is its high clonogenicity, defined by colony-forming unit-fibroblast, CFU-F^20^,^21^. In a 96-well colony formation assay, CFU-F was measured by the negative linear relationship between the seeding density (cell number) and the incidence of no colony formation (% negative response)^20^,^21^. The frequency of CFU-Fs for iMSCs was 1 per 7 cells, which was 16-fold higher than the fibroblasts (1 per 112 cells) (Fig. 1C), suggesting a much higher clonogenicity in iMSCs. The iMSCs and BMMSCs also exhibited much stronger clonogenicity than fibroblasts in the traditional colony-forming assay (Supplementary Figure S1).

### iMSCs have similar molecular signatures to BMMSCs

We next investigated the molecular mechanism of the multipotency induced by our chemical cocktail. One of Yamanaka factors, the pluripotent transcription factor OCT4, has been indicated to contribute to the multipotency of MSCs^22^,^23^. OCT4 was up-regulated in iMSCs to a level similar to BMMSCs as compared to fibroblasts (Fig. 1D), suggesting that iMSCs may be induced through the action of OCT4.

To further compare the molecular signatures, microarray analysis was performed with three parental fibroblasts (a neonatal foreskin fibroblasts CRL2097 and two adult abdominal skin fibroblasts DF440547 and DF443480), three iMSCs from these fibroblasts, and BMMSCs. We also retrieved the expression information of one BMMSC from the publicly available Gene Expression Omnibus (GEO) database. Principal component analysis (PCA) of stemness genes revealed that iMSCs derived from dermal fibroblasts were similar to each other, while the parental fibroblasts and BMMSCs were quite different (Fig. 1E, Supplementary Table S2). Of note, these iMSCs were more similar to BMMSCs when compared to their parental fibroblasts (Fig. 1E). Gene Ontology (GO) analysis revealed that the genes up-regulated in iMSCs and BMMSCs were related to developmental process and cell differentiation, while the genes down-regulated were related to immune system process (Supplementary Figure S2). These data suggest that our chemical cocktail is able to convert fibroblasts to a multipotent cell state that is similar to BMMSCs at the molecular level.

### The conversion of fibroblasts into SSEA-4^high^PODXL^high^ cells is reproducible

The chemical cocktail (6C+3GF) consistently induced the conversion with an efficiency ranging from 26.4–64.0% in ten experiments (mean = 38%) (Supplementary Figure S3). These iMSCs could be maintained and expanded in regular culture medium in the absence of small molecules for at least 8 passages and still express SSEA-4 and PODXL (Supplementary Figure S4). Only the SSEA-4^high^PODXL^high^ cells, but not SSEA-4^low^PODXL^low^ cells, were able to efficiently differentiate into osteoblasts and adipocytes (Supplementary Figure S5).

### iMSCs express all traditional MSC markers

Next, we tested if these SSEA-4^high^PODXL^high^ iMSCs express traditional MSC markers by flow cytometry. The marker expression profiles of iMSCs were nearly identical to BMMSCs (expressing CD90, CD44, CD73, and CD105 surface markers, but not CD11b, CD19, CD34, CD45, and HLA-DR) (Supplementary Figure S6), fulfilling the criteria of MSC markers defined by the ISCT^5^. In contrast to the homogenous marker expression in iMSCs, the expression of these markers was heterogeneous in different sources of fibroblasts. Compare to iMSCs and BMMSCs, foreskin fibroblasts (CRL2097) expressed low level of CD105 (Supplementary Figure S6A), while abdominal fibroblasts (DF443480) expressed low levels of CD90 and CD44 (Supplementary Figure S6B). Both of these two fibroblasts slightly expressed CD11, CD19, CD34, CD45 or HLA-DR (more profound in CRL2097), while these markers were negative in iMSCs/BMMSCs (Supplementary Figure S6). In summary, iMSCs have a similar surface marker profile to BMMSCs that is evidently different from the primary fibroblasts. The microarray (Fig. 1E) and surface marker data (Supplementary Figure S6) both suggest that our chemical cocktail can induce distinct fibroblasts to a more homogenized multipotent cell state.

### iMSCs are multipotent cells similar to BMMSCs

**Figure 2:**
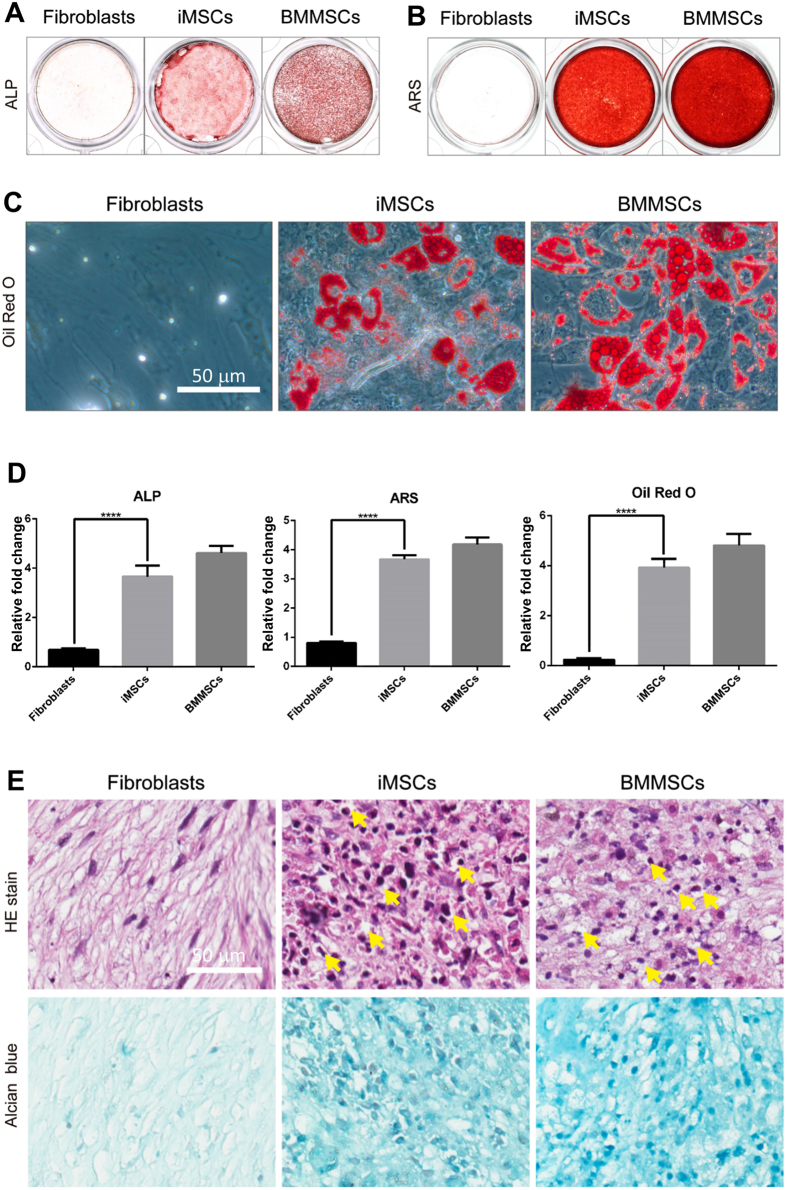
iMSCs derived from dermal fibroblasts CRL2097 are multipotent and can differentiate into osteoblasts, adipocytes, and chondrocytes. (**A**) To detect early osteogenesis, fibroblasts (CRL2097), iMSCs derived from CRL2097 (iMSCs), and BMMSCs were cultured in osteogenic induction medium for 10 days, and the alkaline phosphatase (ALP) activity assay was performed. (**B**) To detect late osteogenesis, Alizarin Red S staining (ARS) was performed at day 21. (**C**) To detect adipogenesis, fibroblasts CRL2097, iMSCs, and BMMSCs were cultured in adipogenic induction medium for 21 days and then stained with Oil Red O. Scale bar: 50 μm. (**D**) The quantification results of the ALP activity, the Alizarin Red S assay, and the Oil Red O staining (n = 6). ****p < 0.0001. (**E**) The lacunae structure (Hematoxylin and eosin staining, HE) and proteoglycans of cartilage (Alcian Blue staining) were examined to evaluate the ability of iMSCs to differentiate into chondrocytes at day 21. The lacunae are marked by the yellow arrow. Scale bar, 50 μm.

To investigate if iMSCs are multipotent, we examined their ability to differentiate into osteoblasts, adipocytes, and chondrocytes. Alkaline phosphatase (ALP) activity and Alizarin Red S staining (ARS) were examined to determine osteogenesis ability. ALP activity is required for bone formation in early osteogenesis; and ARS reveals the extent of calcium deposition, which is required for bone matrix formation in late osteogenesis. After osteogenic induction, iMSCs induced from fibroblasts CRL2097 exhibited ALP activity at day 10 and ARS at day 21 to an extent comparable to that of primary BMMSCs. In contrast, primary fibroblasts could not differentiate into osteoblasts (Fig. 2A,B and D). To determine adipogenesis ability, these iMSCs were cultured in adipogenic induction medium for 21 days, and the lipid drops were then stained with Oil Red O. BMMSCs and iMSCs, but not fibroblasts, efficiently formed lipid drops (Fig. 2C and D). Finally, for chondrogenesis, iMSCs and BMMSCs instead of fibroblasts efficiently differentiated into chondrocytes (Fig. 2E). The extent of chondrogenesis was judged by the presence of lacunae structures in cartilage revealed by hematoxylin-eosin staining and proteoglycans shown by Alcian blue staining (Fig. 2E). Thus, the chemical cocktail can induce fibroblasts CRL2097 to reprogram into functional multipotent iMSCs. We also examined iMSCs derived from dermal fibroblasts of two additional donors (DF440547 and DF443480) and again found that iMSCs exhibited the ability to further differentiate into osteoblasts, adipocytes, and chondrocytes to a degree comparable to BMMSCs (Supplementary Figure S7). Thus, our chemical cocktail can effectively convert fibroblasts into functional, multipotent MSC-like cells.

### iMSCs, like BMMSCs, markedly decrease the fatality of endotoxin-induced acute lung injury in a mouse model and demonstrate anti-inflammation ability

**Figure 3:**
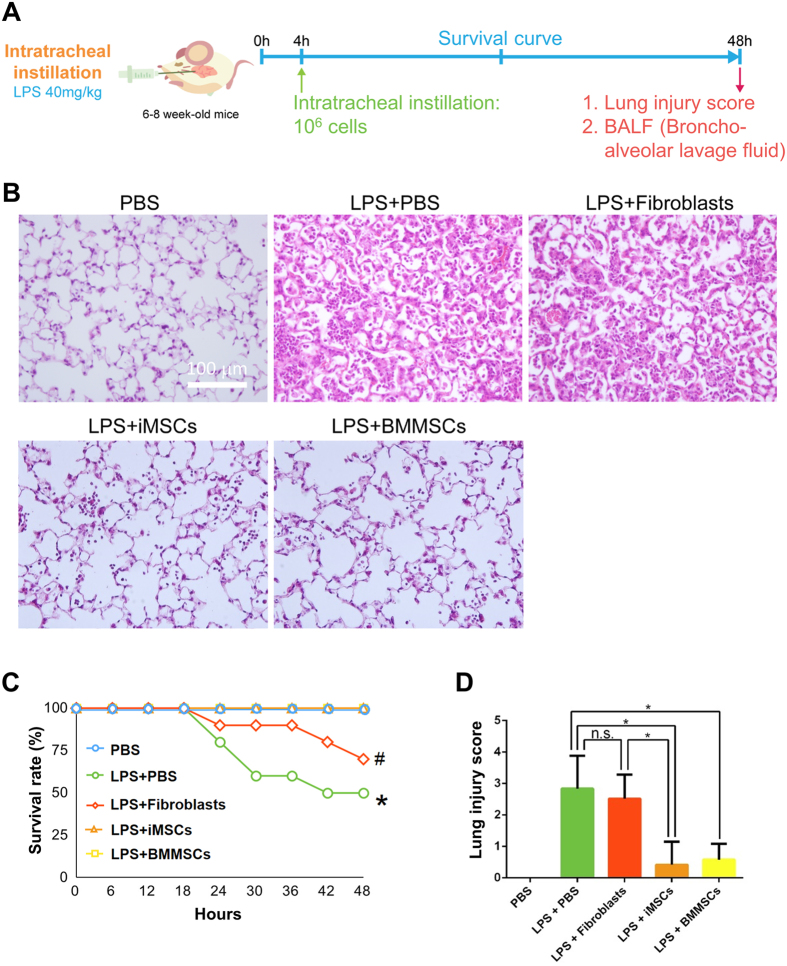
iMSCs, like BMMSCs, significantly ameliorate LPS-induced lung inflammation in mice. (**A**) The experimental scheme for inducing acute lung injury in mice using intratracheal injections of LPS. (**B**) iMSCs and BMMSCs significantly ameliorate lung inflammation. Representative lung histology at 48 h after LPS-induced acute lung injury. Scale bar: 100 μm. (**C**) iMSCs, like BMMSCs, efficiently improve the survival rate of LPS-induced acute lung injury. The results are expressed as a percentage of survival (n = 10–12 per group). *p < 0.05, LPS + PBS V.S. (PBS, LPS + iMSCs, or LPS + BMMSCs). ^#^*p* < 0.05, LPS + Fibroblasts vs. (PBS, LPS + iMSCs, or LPS + BMMSCs). (**D**) The injury score of LPS-induced acute lung injury and the quantification of the histology at 48 h after LPS-induced acute lung injury. All sections were quantified after digital slide scanning of the whole slide. n = 10–12 each group. The details of calculation were described in method. The injury score used the following criteria: 0, no injury; 1, 25% injury in the field; 2, 50% injury in the field; 3, 75% injury in the field; and 4, diffuse lung injury. *p < 0.05.

**Figure 4:**
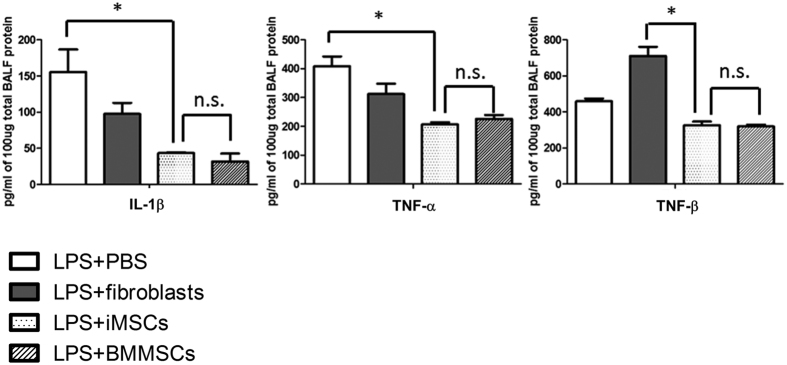
Low levels of proinflammatory cytokines detected in the LPS-induced lung injury mice injected with iMSCs/BMMSCs. After 4 hours, LPS-treated mice were intratracheal instilled with PBS, fibroblasts, iMSCs, or BMMSCs. The expression levels of indicated proinflammatory cytokines in BALF were detected by Multiplex bead array assay. The levels of IL-1β, TNF-α, and TNF-β in BALF were quantified in three independent experiments. Data shows in means with ± SEM. *p < 0.05; **p < 0.01; n.s.: not significant.

Next, to examine if iMSCs can be as effective as BMMSCs *in vivo*, an acute lung injury (ALI) mouse model was performed (Fig. 3A). We found that intratracheal administration of iMSCs or BMMSCs 4 hours after lipopolysaccharide (LPS) treatment significantly repressed acute lung injury (Fig. 3B). Of note, in contrast to the 50% death observed in ALI mice treated with LPS and the solvent control, all mice injected with iMSCs or BMMSCs survived (Fig. 3C). These results were supported by histological evidence of acute lung injury and lung injury scores (Fig. 3B and D). In contrast, fibroblasts were not as effective as iMSCs and BMMSCs in the treatment of ALI (Fig. 3B–D). Thus, iMSCs, like BMMSCs, can inhibit LPS-mediated ALI *in vivo*. MSCs are known to possess immunomodulatory functions^7^,^8^,^24^. To test whether iMSCs contribute to the repair via anti-inflammation, we examined the cytokine secretion levels. After 2 days of LPS treatment, the bronchoalveolar lavage fluid (BALF) was collected and multiplex cytokine assay was performed. The levels of pro-inflammatory cytokines IL-1β, TNF-α, and TNF-β were downregulated in iMSC- and BMMSC-treated mice to very similar levels (Fig. 4). Thus, iMSCs can treat the disease as effectively as BMMSCs possibly due to the similar anti-inflammation ability.

We also used GFP-labeled fibroblasts, iMSCs, and BMMSCs to track their *in vivo* localization at different time points and how they may contribute to the lung repair process. After the injection, we did detect GFP-labeled cells reside in the lung during the 4.5-hour-, 24-hour-, and 48-hour-period (Supplementary Figure S8A). We did not detect any GFP-labeled cells in the bone marrow by FACS (Supplementary Figure S8B), indicating that the lung-injected cells did not migrate to bone marrow within 48 hours. The GFP signal and the epithelial marker pan-cytokeratin (pan-CK) did not colocalize in the same cell (Supplementary Figure S8A), suggesting that fibroblasts/iMSCs/BMMSCs may not directly contribute to the alveolar tissues. Altogether, our results revealed the anti-sepsis effects of iMSCs and BMMSCs should be primarily based on their immunomodulatory functions.

### Three to six chemicals are sufficient to generate iMSCs

**Figure 5:**
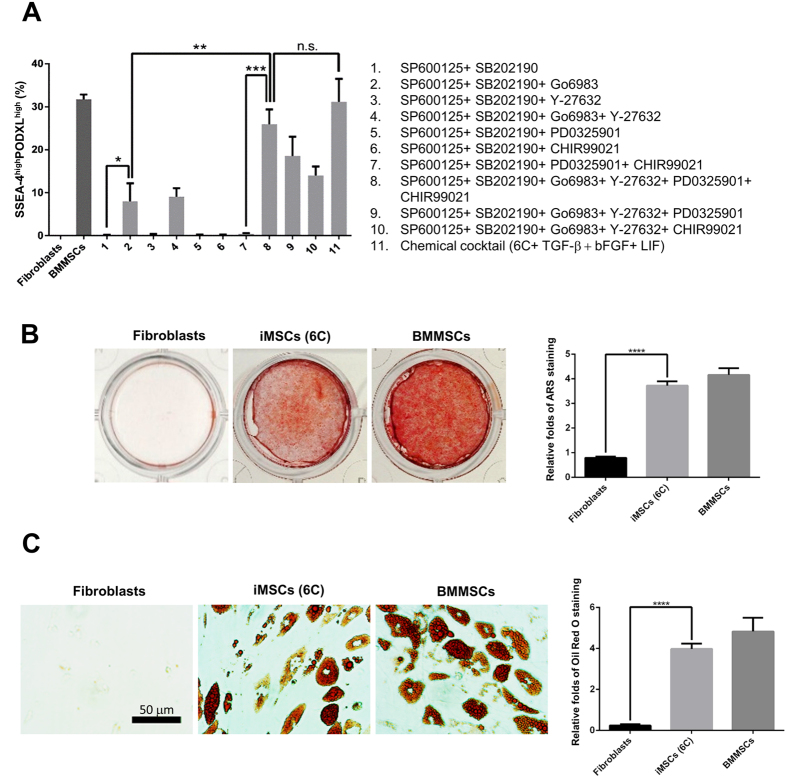
The evaluation of the efficacy of iMSC generation and the optimization of the cocktail compositions. (**A**) The optimization of the cocktail compositions for the conversion of human fibroblasts into iMSCs. The chemical cocktail contained at most six chemical inhibitors: JNKi (SP600125, 10 μM), p38i (SB202190, 10 μM), PKCi (Go6983, 5 μM), ROCKi (Y-27632, 5 μM), ERK1/2i (PD0325901, 1 μM), and GSK3βi (CHIR99021, 3 μM). The SSEA-4^high^PODXL^high^ population was quantified to measure the iMSC conversion efficacy at day 6. Three compounds, SP600125, SB202190, and Go6983, were sufficient for the conversion of human fibroblasts to iMSCs with low efficiency (condition 2). The removal of Y-27632, PD0325901, or CHIR99021 reduced the efficacy of iMSC production (conditions 4, 9, and 10). The cocktail containing all six inhibitors (condition 8) resulted in the most efficacious conversion, with an efficacy comparable to the chemical cocktail (6C+3GF) (condition 11, 6 chemicals with three cytokines). BMMSCs served as the positive control, and fibroblasts served as the negative control. *p < 0.05; **p < 0.01; ***p < 0.001; n.s.: not significant. (**B**) The iMSCs derived from fibroblasts CRL2097 with only 6 chemicals exhibit osteogenesis abilities comparable to those of BMMSCs. Fibroblasts, iMSCs derived with 6 chemicals (6 C), and BMMSCs were cultured in osteoblast-induction medium for 21 days, and then, they were assayed by Alizarin Red staining (ARS) (left panel). The dye was extracted, and ARS was quantified by measuring the optical density (O.D.) at 550 nm (right panel) (n = 6). ****p < 0.0001. (**C**) The iMSCs derived from fibroblasts CRL2097 with only 6 chemicals exhibit adipogenesis abilities comparable to those of BMMSCs. Indicated fibroblasts, iMSCs, and BMMSCs were cultured in adipocyte induction medium for 21 days, and the lipid drops were then stained with Oil Red O (left panel). Scale bar: 50 μm. The dye was extracted, and Oil Red O staining was quantified by measuring the O.D. at 530 nm (right panel) (n = 6). ****p < 0.0001.

Next, we examined whether all chemicals and growth factors (6C+3GF) are required for generating iMSCs. By adding one cytokine at a time, we found that all three growth factors, TGF-β, bFGF, and LIF, were dispensable for iMSC generation (Fig. 5A and Supplementary Figure S9), since six chemicals (6 C) were sufficient to generate iMSCs almost as efficiently as the chemical and growth factor cocktail (6C+3GF). The iMSCs derived from six chemicals (6 C) exhibited the ability to differentiate into osteoblasts (Fig. 5B) and adipocytes (Fig. 5C) as effectively as BMMSCs, suggesting that they are still multipotent. Finally, we tested if all six chemicals were required for the generation of iMSCs. Three chemicals, SP600125, SB202190, and Go6983, were sufficient to generate iMSCs, however at a much lower efficiency (Fig. 5A, condition 2). The removal of any one of these compounds from the six chemical cocktail (6C) reduced the efficiency of iMSC production (Fig. 5A).

## Discussion

To avoid (1) insertional mutagenesis by virus infection or plasmid transfection, (2) the tedious processes of virus preparation, and (3) repeated transfection/transduction, the use of chemical/growth factors to convert fibroblasts has drawn substantial attention recently. To date, chemical/growth factor conversion has been performed in fibroblasts to generate iPSCs (40–60 days)^16^,^25^, neuron cells (21–28 days)^13^, neuron progenitor cells (~20 days)^26^, Schwann cells (>27 days)^27^, cardiomyocytes (24 days)^15^, etc. All of these cell types have also been successfully generated from fibroblasts by a combination of transcription factors^28^,^29^,^30^,^31^,^32^. A recent study has reported that transduction of recombinant Yamanaka factors (SOX2, OCT4, KLF4, and c-MYC) can convert fibroblasts into induced pluripotent mesenchymal stem cells (iPMSCs) in 30 days with the ability to form teratomas^33^. However, the protocols to convert fibroblasts into MSC-like cells by MSC-specific transcription factors have not been reported due to the lack of master regulators.

**Figure 6:**
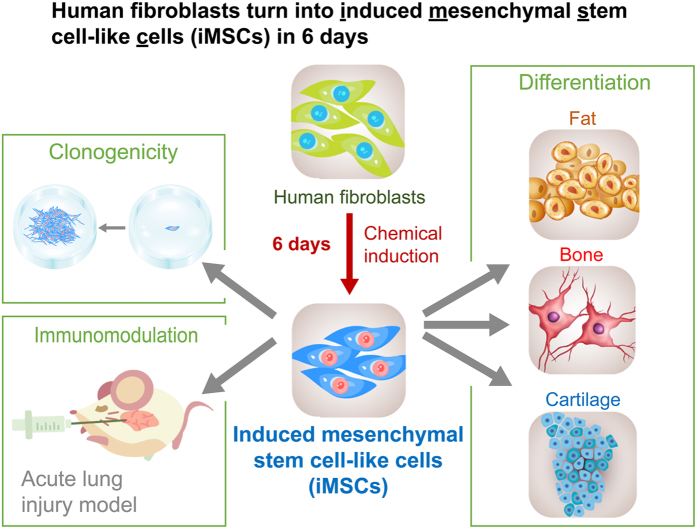
The graphical abstract of chemically induced human MSCs from fibroblasts. The chemical cocktail with six chemical inhibitors with or without three growth factors efficiently and robustly generated iMSCs from primary dermal fibroblasts in six days. iMSCs are multipotent and can further differentiate into osteoblasts (bone lineage), adipocytes (fat lineage), and chondrocytes (cartilage lineage). iMSCs also markedly increased the clonogenicity and decreased the fatality of endotoxin-induced acute lung injury in a mouse model.

In this study, we established the first method to chemically induce MSCs from fibroblasts in six days, which might be one of the fastest chemical conversion protocols of fibroblasts to date. Our data reveal that the cocktail containing six chemical inhibitors (SP600125, SB202190, Go6983, Y-27632, PD0325901, CHIR99021) with or without and three growth factors (TGF-β, bFGF, and LIF) can efficiently generate functional iMSCs from human primary dermal fibroblasts within 6 days (Fig. 6).

For the small molecules in the chemical cocktail that is capable of reprogramming human fibroblasts into iMSCs, all have been suggested to have the possibility to promote stemness by repressing differentiation or promoting stem cell expansion. For example, SB202190 is a specific inhibitor of p38 signaling. The p38 pathway is critical for inducing chondrogenesis, osteoblastogenesis, and neuronal differentiation of MSCs^34^. SP600125 is a JNK inhibitor, which represses the osteogenesis of MSCs^35^,^36^. Go6983 is a PKC inhibitor, which downregulates the osteogenic and cardiogenic differentiation of MSCs^34^,^37^. Y27632 is a ROCK inhibitor that keeps the proliferation of MSCs^38^. ROCK signals can also promote osteogenic, tenogenic, and adipogenic differentiation^39^. PD0325901 is able to inhibit ERK1/2 pathway, which blocks MSC differentiation into bone, fat, and cartilage^40^,^41^,^42^. CHIR99021 is a GSK-3β inhibitor widely used in stem cell reprogramming to support self-renewal ability^34^,^43^,^44^. It is reasonable that combinations of these small molecules can convert fibroblasts into functional, multipotent iMSCs.

The six chemicals and three growth factors (SB202190, SP600125, Go6983, Y27632, PD0325901, CHIR99021, LIF, TGFβ1, bFGF) we used also happened to be included as a part of the components in the naïve human stem cell medium (NHSM)^45^. NHSM can promote the dedifferentiation of human pluripotent stem cells from the ground stage to the naïve stage^45^,^46^, and requires at least 9 factors (LIF, TGFβ1, bFGF, insulin, 2-mercaptoethanol, PD0325901, CHIR99021, SP600125, and SB203580), while the use of 11 factors (LIF, TGFβ1, bFGF, insulin, 2-mercaptoethanol, Y27632, Go6983, PD0325901, CHIR99021, SP600125, and SB203580) might have the best efficiency^45^. Since both NHSM and our cocktail support dedifferentiation, there may be some overlapping signals that trigger the conversion of cell state. However, since only three chemicals (SP600125, SB202190, and Go6983) are sufficient for iMSC generation, and the endogenous states of the parental cell types are also obviously different (fibroblasts vs. ESCs), there must also be some unique signals in the conversion processes (iMSCs vs. naïve stem cells).

Although there is a lack of defined master regulator(s) in MSCs, several studies have indicated that pluripotency markers of ESCs, such as OCT4 and NANOG, might play critical roles in maintaining the potency and proliferative ability of MSCs. Meng, *et al*. reported that ectopic expression of OCT4 with GSK inhibitor, CHIR99021, could convert the human hematopoietic stem cells into iMSCs^47^. Besides, Tsai *et al*. and Piccinato *et al*. also indicated that self-renewal and differentiation ability of MSCs could be upregulated under hypoxia condition with high expression of OCT4 and other factors^22^,^23^. Our chemical induction method might involve similar regulations through OCT4 to promote the multipotency of fibroblasts (Fig. 1D).

Like BMMSCs, iMSCs can differentiate into osteoblasts, adipocytes, and chondrocytes (Fig. 2, Supplementary Figure S7). Moreover, they markedly attenuate the fatality of endotoxin-induced acute lung injury and decrease the amounts of proinflammatory cytokines in the lung in a mouse model (Figs 3 and 4). iMSCs also share similar molecular signatures with BMMSCs (Fig. 1) and fulfill all of the MSC criteria defined by ISCT^5^, including plastic adherence, multipotency (Fig. 2, Supplementary Figure S7), and marker expressions (Supplementary Figure S6). The clonogenicity of iMSCs is also similar to BMMSCs (Fig. 1C, Supplementary Figure S1).

Due to the known immunosuppressive ability of MSCs, we applied the well-established endotoxin-induced acute lung injury model as in previous studies to test *in vivo* function of our iMSCs^7^,^48^,^49^,^50^. In those studies, human MSCs were injected into normal mice such as Balb/c^48^ and C57BL/6^49^,^50^ to attenuate lung injury. Our iMSCs demonstrated similar immunomodulatory ability as human BMMSCs in a mouse model through decreasing the amount of proinflammatory cytokines within 48 hours (Figs 3 and 4). Previous studies demonstrated that murine bone marrow MSC can integrate into murine lung epithelium in bleomycin and naphthalene injury models in 2–14 days^51^,^52^, while human core blood MSCs (CB-MSCs) can differentiate into lung epithelial-like cells in NOD-SCID mice in 14 days^53^. We also intratracheally instilled GFP-labeled human fibroblasts, iMSCs, and BMMSCs into mouse lung at 4-hour-post-injury, and lung sections were analyzed at 4.5-, 24-, and 48-hour post-injury with confocal microscopy. We did not observe pan-cytokeratin staining in GFP-labeled cells (Supplementary Figure S8), suggesting that the injected cells did not differentiate into lung epithelial cells during the 48-hour experimental period.

For isolation of iMSCs, we selected two functional markers of MSCs, SSEA-4, and PODXL, which have been reported to enrich MSCs with strong multipotency and expansion ability^9^,^10^,^11^. Indeed, fibroblasts expressed very low levels of SSEA-4 and PODXL (0.1–0.3% SSEA-4^high^PODXL^high^) (Fig. 1B; Supplementary Figure S5) and had no differentiation ability into osteoblasts, adipocytes, and chondrocytes (Figs 2 and 5; Supplementary Figures S5 and S7). The iMSCs enriched by SSEA-4 and PODXL were functionally comparable to BMMSCs in terms of their clonogenicity, multipotency and the ability to suppress LPS-lethality in mice (Fig. 6). They also expressed similar molecular signatures and markers with BMMSCs^5^ (Fig. 1B,D and E; Supplementary Figure S6). Therefore, using both SSEA-4 and PODXL as markers, to our knowledge, is successful and arguably the best method to isolate functional iMSCs.

The morphology of iMSCs was similar to BMMSCs and fibroblasts without senescence after the chemical induction. To obtain sufficient amounts of iMSCs for differentiation and mouse anti-sepsis assay, we expanded the induced cells in normal MSC culture medium for 6–8 passages. These iMSCs still expressed functional markers SSEA-4 and PODXL and maintained the morphology and proliferation ability at passage 8, suggesting that they are relatively stable and suitable for clinical applications.

To date, MSCs have been approved to treat GvHD in Canada and New Zealand, and degenerative arthritis and anal fistula in Korea^8^. In addition, MSCs have shown beneficial effects in clinical trials focusing on the treatment of diabetes, multiple sclerosis, kidney transplantation, Crohn’s disease, systemic lupus erythematosus (SLE), and ulcerative colitis^8^. Until now, almost no safety concern has been reported with the use of MSCs in the clinical trials^54^ (www.clinicaltrials.gov). Thus, chemically induced MSCs hold great promise for the treatment of acute lung injury (ALI)^7^, graft-versus-host disease (GvHD)^24^, and multiple diseases (www.clinicaltrials.gov). Since the components of the conversion medium are well-defined and serum-free, our chemical reprogramming method allows standardization and reproducibility with great ease. The method also does not involve any processes that may lead to insertional mutagenesis, therefore we envision it to profoundly benefit the study of stem cell biology and regenerative medicine.

## Materials and Methods

All methods were performed in accordance with the relevant guidelines and regulations.

### Reagents

All culture media (unless otherwise specified) were purchased from Invitrogen (Carlsbad, CA, USA). All chemicals in the cocktails were purchased from LC Laboratories (Woburn, MA, USA), except Go6983, which was obtained from TOCRIS (Bristol, UK) (Supplementary Table S1). All recombinant proteins were purchased from Peprotech (Rocky Hill, NJ, USA) Supplementary Table S3). All other chemicals were purchased from Sigma-Aldrich (St. Louis, MO, USA).

### Cell culture

All experiments with primary human cells were carried out in accordance with relevant guidelines and regulations. All experimental protocols were approved by Human Subject Research Ethics, Academia Sinica (Taipei, Taiwan) (ID: AS-IRB03–105122). Human primary neonatal foreskin fibroblasts (CRL2097) started from passage 4 after isolation were purchased from the ATCC (Manassas, VA, USA) and cultured in Dulbecco’s Modified Eagle Media-high glucose (DMEM-HG) medium with 10% fetal bovine serum (FBS) (HyClone, Logan, UT, USA). Primary adult abdominal skin fibroblasts derived from two adult females (DF440547 and DF443480), who are 42 and 56 years old, respectively (LONZA, Basel, Switzerland). These cells were cultured in DMEM-HG with 10% FBS and started in culture from passage 1. Human primary bone marrow mesenchymal stem cells (BMMSCs) were purchased from LONZA and were cultured with Dulbecco’s Modified Eagle Media-low glucose (DMEM-LG) medium containing 10% FBS. All cells were cultured at 37 °C under 5% CO_2_. All primary cells were cultured within 10 passages after isolation. The informed consent forms were obtained from all the subjects by ATCC and Lonza.

### Generation of iMSCs

For iMSC generation, primary fibroblasts maintained for less than 10 passages were cultured in DMEM-HG for 2 days. The culture medium was then replaced with the chemical cocktail medium (6C+3GF) (Supplementary Table S3) or different combinations for 6 days. Then, cell sorting was performed. Cells were cultured at 37 °C under 5% CO_2_. To obtain sufficient amounts of iMSCs for differentiation assay and mouse anti-sepsis assay, the cells were expanded to 6–8 passages.

### Flow cytometry and cell sorting

For cell surface marker analysis, BMMSCs, iMSCs, and fibroblasts were incubated with FITC-conjugated anti-human SSEA-4 (clone MC-813-70; eBiosciences, San Diego, CA, USA) and PE-conjugated anti-human PODXL (clone B34D1.3; eBiosciences) antibodies. The cells were then analyzed by FACSCanto (Becton Dickinson, Franklin Lakes, NJ, USA). SSEA-4^high^ and PODXL^high^ cells were isolated using a cell sorter (FACS Aria^TM^ II, BD Biosciences). The data was further analyzed by FACSDiva software (BD Biosciences).

### CFU-F negative linear relationship assay

In this assay, the decrease of seeding cell density increases the incidence of wells with no colonies. Sorted cells were seeded into 96-well plates containing 8 cell-dose groups: 0, 5, 10, 20, 40, 80, 160, 320 sorted cells per well. After incubation in optimal condition for 14 days, the wells with no colony were counted for each condition. The seeding cell number in which 37% of the wells are negative for colony formation is defined as the cell concentration of one CFU-F^20^,^21^.

### Microarray analysis

RNA was purified using an RNeasy kit (Qiagen, Hilden, Germany). Samples were prepared for array hybridization using the human Affymetrix 3’ IVT Express kit with GeneChip^®^ Human Genome U133A 2.0 chips following the manufacturer’s protocol (Affymetrix, Santa Clara, CA, USA). The array results were analyzed using the Gene Spring GX 12.6 software (Agilent Technologies, Santa Clara, CA, USA). The microarray profiling of BMMSC_2 was described previously in the Pubmed GEO database (GSM1533333), and all other array data were uploaded to the Pubmed GEO database (GSE72693).

### Osteogenic differentiation

Human primary BMMSCs and iMSCs were cultured in DMEM-LG medium with 10% FBS. Fibroblasts were cultured in DMEM-HG with 10% FBS. To induce differentiation, cells (1 × 10^4^ cells/cm^2^) were cultured with osteogenic induction medium (90% DMEM-HG, 10% FBS, 0.1 μM dexamethasone, 10 mM beta-glycerophosphate, and 0.05 mM L-ascorbic acid phosphate). The media were replaced twice per week during differentiation.

### Alkaline phosphatase activity assay

After 10 days of osteogenic differentiation, cells were fixed with 4% PFA in PBS for 3 minutes. Alkaline phosphatase (ALP) staining was performed using alkaline phosphatase kits in accordance with the manufacturer’s instruction (Sigma-Aldrich). For the quantification of ALP activity, the cells were washed twice with PBS and incubated with the ALP substrate p-nitrophenyl phosphate (pNPP) at room temperature for 5–20 minutes. The absorbance at an optical density (O.D.) at 405 nm was measured.

### Alizarin Red S staining

After 21 days of osteogenic differentiation, cells were fixed with ice-cold 70% ethanol at −20 °C for 1 hour and then washed with PBS. The cells were then stained with 40 mM Alizarin Red S (ARS) (pH 4.2) for 10 minutes and subsequently washed five times with ddH_2_O before being air dried. For quantification, the cells were incubated with 1 mL of cetylpyridinium chloride buffer for 1 h to extract ARS, and the O.D. at 550 nm was then recorded.

### Adipogenic differentiation

For adipogenic induction, cells were cultured in adipogenic induction medium (Biological Industries, Kibbutz Beit-Haemek, Israel), which was replaced twice per week during the 21-day differentiation period.

### Oil Red O staining

Cells were fixed with 4% PFA for 1 hour, washed with 60% isopropanol, and then air dried. The lipid vesicles were stained with Oil Red O staining medium (30 ml of 0.5% oil red solution in 2-propanol diluted with 20 ml of water) for 10 minutes and then washed with distilled water. For quantification, Oil Red O was extracted with isopropanol, and the absorbance at 530 nm was measured.

### Chondrogenic differentiation

For chondrogenic differentiation, BMMSCs, iMSCs, and fibroblasts (2.5 × 10^5^ cells) in separate 15-mL tubes were centrifuged at 500 g for 10 minutes. The pelleted cells were then incubated with chondrogenic induction medium (Biological Industries). The cells formed a spherical aggregate after overnight incubation. The cells were continuously induced for 21 days, and paraffin sections were taken to analyze the samples. After deparaffinization, the slides were stained with Hematoxylin-eosin or an Alcian blue solution.

### Alcian blue staining

The pelleted cells were embedded in paraffin. After sectioning, slides were deparaffinized with xylene and hydrated with distilled water. After incubation of slides in 3% acetic acid for 3 minutes, the slides were stained with a 1% Alcian blue solution (in 3% acetic acid, pH 2.5) for 30–45 minutes. The slides were then washed with water for 2 minutes, dehydrated with xylene, and mounted with mounting solution (Thermo Fisher Scientific, Waltham, MA, USA).

### Endotoxin-induced acute lung injury in mice

All animal experimental procedures and methods were performed in accordance with the relevant guidelines and regulations. All animal protocols were approved by Academia Sinica Institutional Animal Care and Utilization Committee (Protocol ID: 15–07–840). BALB/c female mice (6–8 weeks, National Laboratory Animal Center, Taipei) were first anesthetized with Tiletamine/Zolazepam (25 mg/kg) and xylazine (10 mg/kg) via an intraperitoneal route. Acute lung injury (ALI) was then induced by the intratracheal (i.t.) instillation of 40 mg/kg lipopolysaccharides (LPS) purified from *E. coli* O55:B5 (Sigma-Aldrich) or 100 μL PBS. At four hours after LPS treatment, mice were anesthetized again and then randomly divided into four groups: (1) PBS, (2) human fibroblasts (10^6^ cells in 100 μL PBS), (3) iMSCs (10^6^ cells in 100 μL PBS), and (4) human BMMSCs (10^6^ cells in 100 μL PBS). The survival of mice was followed for 48 h. The survival rate of each group was observed every 6 hours. For the histology analysis and lung injury analysis, the samples were collected before or at 48 hours post injection. The lung sections were fixed and stained. The whole lung area was scanned by Aperio ScanScope XT machine (Leica, Wetzlar, Germany). Then, Aperio ImageScope program (Leica) was used to analyze the total lung section under 4× zoom by adding 100 μm × 100 μm grid. Images of each lung section were further quantified by counting lung section area and inflammatory area. The injury area, which has alveolar and interstitial inflammation (immune cell number >5 in a 100 μm × 100 μm grid), was counted. The ratio of injury area in lung section is calculated as (injury area)/(lung section area). Injury score was determined by the following criteria: 0, no injury; 1, 25% injury; 2, 50% injury; 3, 75% injury; 4, 100% injury.

### Multiplex bead array assay

To quantify the pro-inflammatory cytokine secretion levels, the antibody-coated beads in a high sensitivity MILLIPLEX kit (Merck Millipore, Darmstadt, Germany) was used in accordance with the manufacturer’s protocol. To measure the amounts of selected cytokines (IL-1β, TNF-α, and TNF-β), BALF from LPS-induced acute lung injury mice injected with PBS, fibroblasts, iMSCs, and BMMSCs respectively were collected. Then different interleukins antibody-coated beads were incubated with 100 μg total BALF protein for 16 hours and further analyzed by Luminex 200 (Merck)^55^.

### Statistical analysis

All statistical data are presented as the mean ± standard deviation (S.D.) of at least three biological replicates. Statistically significant differences were assessed by Student’s unpaired two-tailed *t*-test, where *p*-value < 0.05 was considered a significant difference.

## Additional Information

**Accession codes:** The microarray data have been deposited in GEO database under the accession code GSE72693.

**How to cite this article**: Lai, P.-L. *et al*. Efficient Generation of Chemically Induced Mesenchymal Stem Cells from Human Dermal Fibroblasts. *Sci. Rep.* **7**, 44534; doi: 10.1038/srep44534 (2017).

**Publisher's note:** Springer Nature remains neutral with regard to jurisdictional claims in published maps and institutional affiliations.

## Supplementary Material

Supplementary Information

## References

[b1] Pittenger M. F. . Multilineage potential of adult human mesenchymal stem cells. Science 284, 143–147 (1999). 10102814 10.1126/science.284.5411.143

[b2] Xu R. . hiPS-MSCs differentiation towards fibroblasts on a 3D ECM mimicking scaffold. Sci Rep 5, 8480, doi: 10.1038/srep08480 (2015). 25684543 PMC4329554

[b3] Wang Y., Chen X., Cao W. & Shi Y. Plasticity of mesenchymal stem cells in immunomodulation: pathological and therapeutic implications. Nat Immunol 15, 1009–1016, doi: 10.1038/ni.3002 (2014). 25329189

[b4] Karp J. M. & Leng Teo G. S. Mesenchymal stem cell homing: the devil is in the details. Cell Stem Cell 4, 206–216, doi: 10.1016/j.stem.2009.02.001 (2009). 19265660

[b5] Dominici M. . Minimal criteria for defining multipotent mesenchymal stromal cells. The International Society for Cellular Therapy position statement. Cytotherapy 8, 315–317, doi: 10.1080/14653240600855905 (2006). 16923606

[b6] Menard C. & Tarte K. Immunoregulatory properties of clinical grade mesenchymal stromal cells: evidence, uncertainties, and clinical application. Stem Cell Res Ther 4, 64, doi: 10.1186/scrt214 (2013). 23742637 PMC3706914

[b7] Fang X. . Human Mesenchymal Stem (Stromal) Cells Promote the Resolution of Acute Lung Injury in Part through Lipoxin A4. J Immunol 195, 875–881, doi: 10.4049/jimmunol.1500244 (2015). 26116507

[b8] Gao F. . Mesenchymal stem cells and immunomodulation: current status and future prospects. Cell Death Dis 7, e2062, doi: 10.1038/cddis.2015.327 (2016). 26794657 PMC4816164

[b9] Gang E. J., Bosnakovski D., Figueiredo C. A., Visser J. W. & Perlingeiro R. C. SSEA-4 identifies mesenchymal stem cells from bone marrow. Blood 109, 1743–1751, doi: 10.1182/blood-2005-11-010504 (2007). 17062733

[b10] Mihaila S. M. . The osteogenic differentiation of SSEA-4 sub-population of human adipose derived stem cells using silicate nanoplatelets. Biomaterials 35, 9087–9099, doi: 10.1016/j.biomaterials.2014.07.052 (2014). 25123923

[b11] Lee R. H. . The CD34-like protein PODXL and alpha6-integrin (CD49f) identify early progenitor MSCs with increased clonogenicity and migration to infarcted heart in mice. Blood 113, 816–826, doi: 10.1182/blood-2007-12-128702 (2009). 18818395 PMC2630267

[b12] Alt E. . Fibroblasts share mesenchymal phenotypes with stem cells, but lack their differentiation and colony-forming potential. Biology of the cell/under the auspices of the European Cell Biology Organization 103, 197–208, doi: 10.1042/BC20100117 (2011). 21332447

[b13] Hu W. . Direct Conversion of Normal and Alzheimer’s Disease Human Fibroblasts into Neuronal Cells by Small Molecules. Cell stem cell 17, 204–212, doi: 10.1016/j.stem.2015.07.006 (2015). 26253202

[b14] Li X. . Small-Molecule-Driven Direct Reprogramming of Mouse Fibroblasts into Functional Neurons. Cell stem cell 17, 195–203, doi: 10.1016/j.stem.2015.06.003 (2015). 26253201

[b15] Fu Y. . Direct reprogramming of mouse fibroblasts into cardiomyocytes with chemical cocktails. Cell research 25, 1013–1024, doi: 10.1038/cr.2015.99 (2015). 26292833 PMC4559819

[b16] Zhao Y. . A XEN-like State Bridges Somatic Cells to Pluripotency during Chemical Reprogramming. Cell 163, 1678–1691, doi: 10.1016/j.cell.2015.11.017 (2015). 26686652

[b17] Ng F. . PDGF, TGF-beta, and FGF signaling is important for differentiation and growth of mesenchymal stem cells (MSCs): transcriptional profiling can identify markers and signaling pathways important in differentiation of MSCs into adipogenic, chondrogenic, and osteogenic lineages. Blood 112, 295–307, doi: 10.1182/blood-2007-07-103697 (2008). 18332228

[b18] Ma S. . Immunobiology of mesenchymal stem cells. Cell Death Differ 21, 216–225, doi: 10.1038/cdd.2013.158 (2014). 24185619 PMC3890955

[b19] Lin T. & Wu S. Reprogramming with Small Molecules instead of Exogenous Transcription Factors. Stem cells international 2015, 794632, doi: 10.1155/2015/794632 (2015). 25922608 PMC4397468

[b20] Stingl J. . Purification and unique properties of mammary epithelial stem cells. Nature 439, 993–997, doi: 10.1038/nature04496 (2006). 16395311

[b21] Morikawa S. . Prospective identification, isolation, and systemic transplantation of multipotent mesenchymal stem cells in murine bone marrow. J Exp Med 206, 2483–2496, doi: 10.1084/jem.20091046 (2009). 19841085 PMC2768869

[b22] Tsai C. C., Su P. F., Huang Y. F., Yew T. L. & Hung S. C. Oct4 and Nanog directly regulate Dnmt1 to maintain self-renewal and undifferentiated state in mesenchymal stem cells. Mol Cell 47, 169–182, doi: 10.1016/j.molcel.2012.06.020 (2012). 22795133

[b23] Piccinato C. A., Sertie A. L., Torres N., Ferretti M. & Antonioli E. High OCT4 and Low p16(INK4A) Expressions Determine *In Vitro* Lifespan of Mesenchymal Stem Cells. Stem Cells Int 2015, 369828, doi: 10.1155/2015/369828 (2015). 26089914 PMC4454755

[b24] Le Blanc K. . Treatment of severe acute graft-versus-host disease with third party haploidentical mesenchymal stem cells. Lancet 363, 1439–1441, doi: 10.1016/S0140-6736(04)16104-7 (2004). 15121408

[b25] Hou P. . Pluripotent stem cells induced from mouse somatic cells by small-molecule compounds. Science 341, 651–654, doi: 10.1126/science.1239278 (2013). 23868920

[b26] Cheng L. . Generation of neural progenitor cells by chemical cocktails and hypoxia. Cell Res 24, 665–679, doi: 10.1038/cr.2014.32 (2014). 24638034 PMC4042166

[b27] Thoma E. C. . Chemical conversion of human fibroblasts into functional Schwann cells. Stem Cell Reports 3, 539–547, doi: 10.1016/j.stemcr.2014.07.014 (2014). 25358782 PMC4223700

[b28] Caiazzo M. . Direct generation of functional dopaminergic neurons from mouse and human fibroblasts. Nature 476, 224–227, doi: 10.1038/nature10284 (2011). 21725324

[b29] Meng F. . Induction of fibroblasts to neurons through adenoviral gene delivery. Cell Res 22, 436–440, doi: 10.1038/cr.2011.185 (2012). 22105483 PMC3271579

[b30] Ieda M. . Direct reprogramming of fibroblasts into functional cardiomyocytes by defined factors. Cell 142, 375–386, doi: 10.1016/j.cell.2010.07.002 (2010). 20691899 PMC2919844

[b31] Najm F. J. . Transcription factor-mediated reprogramming of fibroblasts to expandable, myelinogenic oligodendrocyte progenitor cells. Nat Biotechnol 31, 426–433, doi: 10.1038/nbt.2561 (2013). 23584611 PMC3678540

[b32] Takahashi K. & Yamanaka S. Induction of pluripotent stem cells from mouse embryonic and adult fibroblast cultures by defined factors. Cell 126, 663–676, doi: 10.1016/j.cell.2006.07.024 (2006). 16904174

[b33] Chen F. . High-efficiency generation of induced pluripotent mesenchymal stem cells from human dermal fibroblasts using recombinant proteins. Stem Cell Res Ther 7, 99, doi: 10.1186/s13287-016-0358-4 (2016). 27473118 PMC4967313

[b34] Song H., Chang W., Song B. W. & Hwang K. C. Specific differentiation of mesenchymal stem cells by small molecules. Am J Stem Cells 1, 22–30 (2012). 23671794 PMC3643383

[b35] Tominaga S., Yamaguchi T., Takahashi S., Hirose F. & Osumi T. Negative regulation of adipogenesis from human mesenchymal stem cells by Jun N-terminal kinase. Biochem Biophys Res Commun 326, 499–504, doi: 10.1016/j.bbrc.2004.11.056 (2005). 15582605

[b36] Kilian K. A., Bugarija B., Lahn B. T. & Mrksich M. Geometric cues for directing the differentiation of mesenchymal stem cells. Proc Natl Acad Sci USA 107, 4872–4877, doi: 10.1073/pnas.0903269107 (2010). 20194780 PMC2841932

[b37] Zhu F., Sweetwyne M. T. & Hankenson K. D. PKCdelta is required for Jagged-1 induction of human mesenchymal stem cell osteogenic differentiation. Stem Cells 31, 1181–1192, doi: 10.1002/stem.1353 (2013). 23404789

[b38] Nakamura K., Yoshimura A., Kaneko T., Sato K. & Hara Y. ROCK inhibitor Y-27632 maintains the proliferation of confluent human mesenchymal stem cells. J Periodontal Res 49, 363–370, doi: 10.1111/jre.12114 (2014). 23834550

[b39] Maharam E. . Rho/Rock signal transduction pathway is required for MSC tenogenic differentiation. Bone Res 3, 15015, doi: 10.1038/boneres.2015.15 (2015). 26509098 PMC4605238

[b40] Gharibi B., Ghuman M. S. & Hughes F. J. Akt- and Erk-mediated regulation of proliferation and differentiation during PDGFRbeta-induced MSC self-renewal. J Cell Mol Med 16, 2789–2801, doi: 10.1111/j.1582-4934.2012.01602.x (2012). 22805337 PMC4118247

[b41] Mei Y. . miR-21 modulates the ERK-MAPK signaling pathway by regulating SPRY2 expression during human mesenchymal stem cell differentiation. J Cell Biochem 114, 1374–1384, doi: 10.1002/jcb.24479 (2013). 23239100

[b42] Fu L. . Stimulation of osteogenic differentiation and inhibition of adipogenic differentiation in bone marrow stromal cells by alendronate via ERK and JNK activation. Bone 43, 40–47, doi: 10.1016/j.bone.2008.03.008 (2008). 18486585

[b43] Li W. . Generation of human-induced pluripotent stem cells in the absence of exogenous Sox2. Stem Cells 27, 2992–3000, doi: 10.1002/stem.240 (2009). 19839055 PMC3780784

[b44] Ying Q. L. . The ground state of embryonic stem cell self-renewal. Nature 453, 519–523, doi: 10.1038/nature06968 (2008). 18497825 PMC5328678

[b45] Gafni O. . Derivation of novel human ground state naive pluripotent stem cells. Nature 504, 282–286, doi: 10.1038/nature12745 (2013). 24172903

[b46] Fang R. . Generation of naive induced pluripotent stem cells from rhesus monkey fibroblasts. Cell Stem Cell 15, 488–496, doi: 10.1016/j.stem.2014.09.004 (2014). 25280221

[b47] Meng X. . Rapid and efficient reprogramming of human fetal and adult blood CD34+ cells into mesenchymal stem cells with a single factor. Cell Res 23, 658–672, doi: 10.1038/cr.2013.40 (2013). 23478301 PMC3641600

[b48] Kim E. S. . Intratracheal transplantation of human umbilical cord blood-derived mesenchymal stem cells attenuates Escherichia coli-induced acute lung injury in mice. Respir Res 12, 108, doi: 10.1186/1465-9921-12-108 (2011). 21843339 PMC3166924

[b49] Bustos M. L. . Activation of human mesenchymal stem cells impacts their therapeutic abilities in lung injury by increasing interleukin (IL)-10 and IL-1RN levels. Stem Cells Transl Med 2, 884–895, doi: 10.5966/sctm.2013-0033 (2013). 24089414 PMC3808203

[b50] Hao Q. . Study of Bone Marrow and Embryonic Stem Cell-Derived Human Mesenchymal Stem Cells for Treatment of Escherichia coli Endotoxin-Induced Acute Lung Injury in Mice. Stem Cells Transl Med 4, 832–840, doi: 10.5966/sctm.2015-0006 (2015). 25999518 PMC4479628

[b51] Rojas M. . Bone marrow-derived mesenchymal stem cells in repair of the injured lung. Am J Respir Cell Mol Biol 33, 145–152, doi: 10.1165/rcmb.2004-0330OC (2005). 15891110 PMC2715309

[b52] Serikov V. B., Popov B., Mikhailov V. M., Gupta N. & Matthay M. A. Evidence of temporary airway epithelial repopulation and rare clonal formation by BM-derived cells following naphthalene injury in mice. Anat Rec (Hoboken) 290, 1033–1045, doi: 10.1002/ar.20574 (2007). 17661377

[b53] Sueblinvong V. . Derivation of lung epithelium from human cord blood-derived mesenchymal stem cells. Am J Respir Crit Care Med 177, 701–711, doi: 10.1164/rccm.200706-859OC (2008). 18063840 PMC2277209

[b54] de Girolamo L. . Mesenchymal stem/stromal cells: a new “cells as drugs” paradigm. Efficacy and critical aspects in cell therapy. Curr Pharm Des 19, 2459–2473 (2013). 23278600 10.2174/1381612811319130015 PMC3788322

[b55] Nold M. F. . IL-37 is a fundamental inhibitor of innate immunity. Nat Immunol 11, 1014–1022, doi: 10.1038/ni.1944 (2010). 20935647 PMC3537119

