# Supplementary material for: Efficient Generation of Chemically Induced Mesenchymal Stem Cells from Human Dermal Fibroblasts
Source: Sci Rep. 2017 Mar 17;7:44534. doi: 10.1038/srep44534 (PMC5356011; doi:10.1038/srep44534)
Supplement: Supplementary Information [file srep44534-s1.doc]

**Supplementary information**

# Efficient Generation of Chemically Induced Mesenchymal Stem Cells from Human Dermal Fibroblasts

Pei-Lun Lai, Hsuan Lin, Shang-Fu Chen, Shang-Chih Yang, Kuo-Hsuan Hung, Ching-Fang Chang, Hsiang-Yi Chang, Frank Leigh Lu, Yi-Hsuan Lee, Yu-Chuan Liu, Hsiao-Chun Huang, and Jean Lu

**
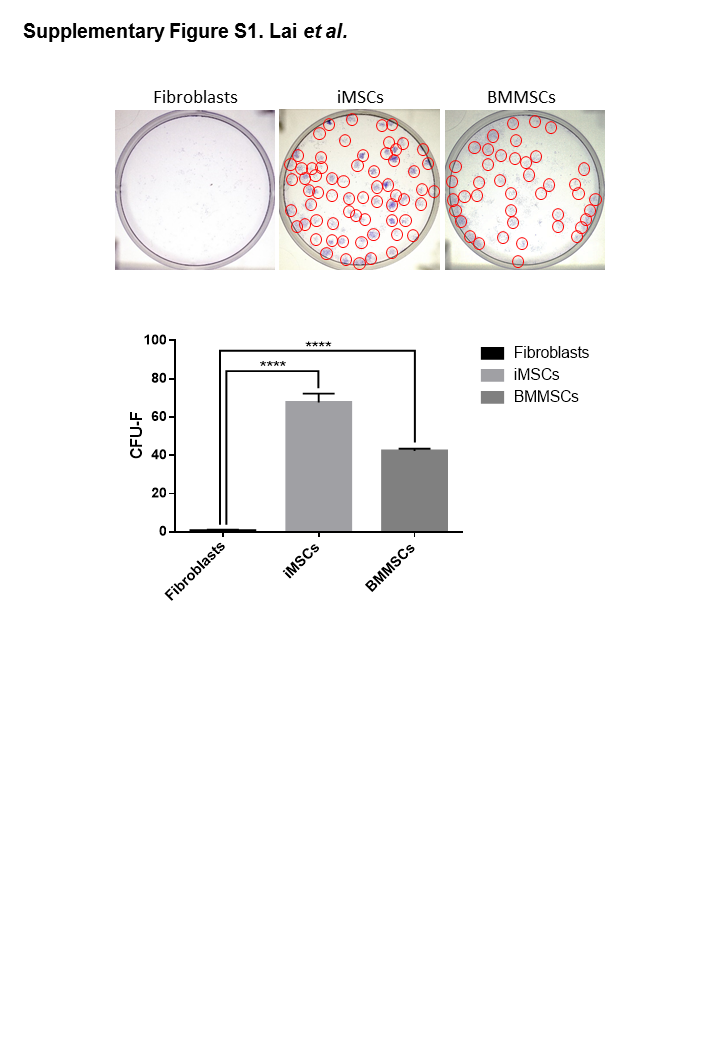
**

**Figure S1. Clonogenicity of fibroblasts, iMSCs, and BMMSCs.** Colony formation assay of fibroblasts, iMSCs, and BMMSCs (500 cells were seeded per well of 6-well plates and incubated 14 days for crystal violet staining) is shown by the whole-well staining in the upper panel. Statistical analysis with three biological replicates is shown in the lower panel (n=3). *****p*<0.0001.

**
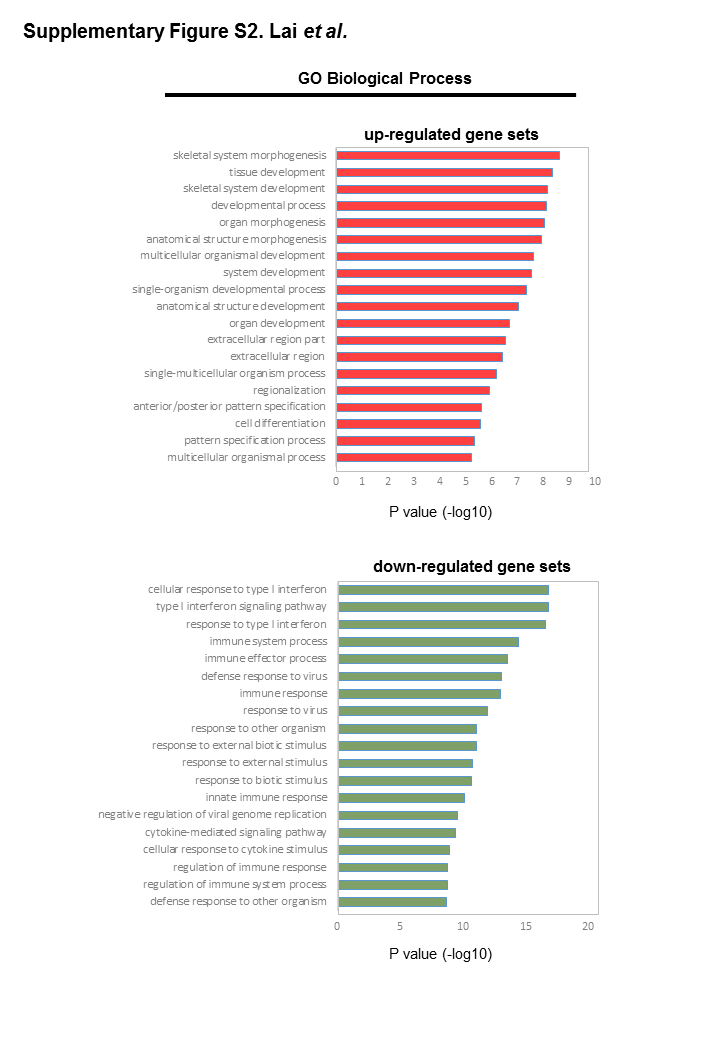
**

**Figure S2. Enrichment of gene ontology (GO) biological processes in iMSCs.** Genes that were at least 2-fold upregulated or downregulated in iMSCs and BMMSCs while compared to fibroblasts were used in the analysis. Top-ranking GO terms of the genes that were up-regulated (upper panel) and down-regulated (lower panel) in iMSCs and BMMSCs were shown.


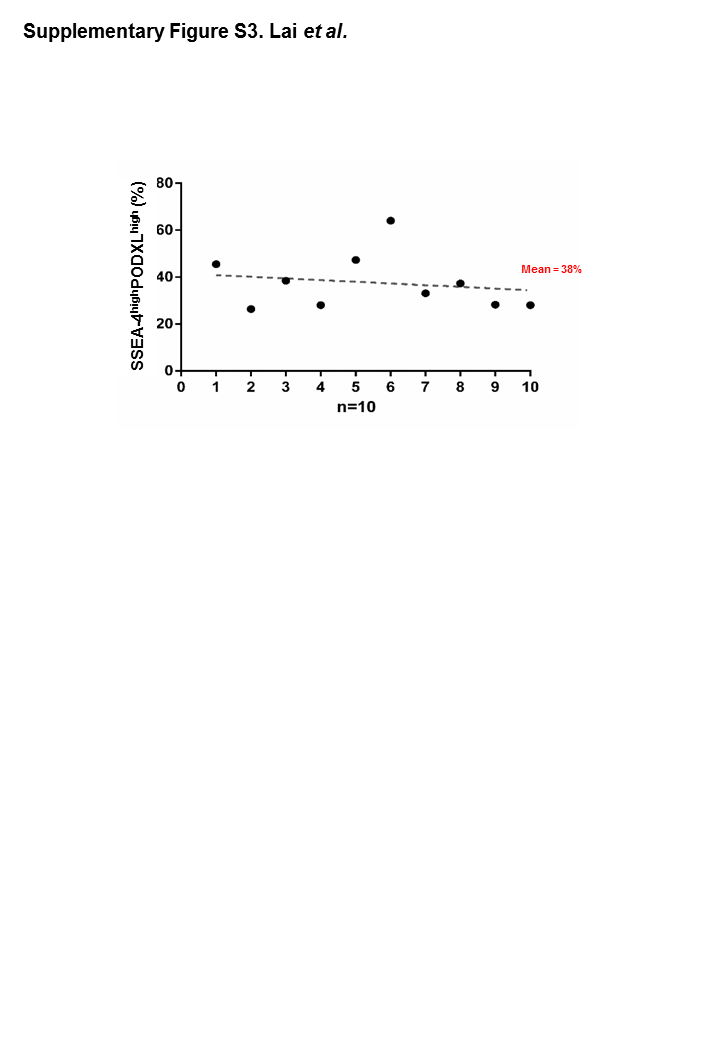
**Figure S3. The chemical cocktail (6C+3GF) reproducibly converted human primary fibroblasts into iMSCs with high efficiency.**

Human dermal fibroblasts were treated with the chemical cocktail for 6 days. Then the cells were subjected to flow cytometry analysis to examine the expression levels of MSC functional markers SSEA-4 and PODXL. Ten independent experiments were performed, and the average iMSC induction rate of the chemical cocktail was shown at 38%.

**
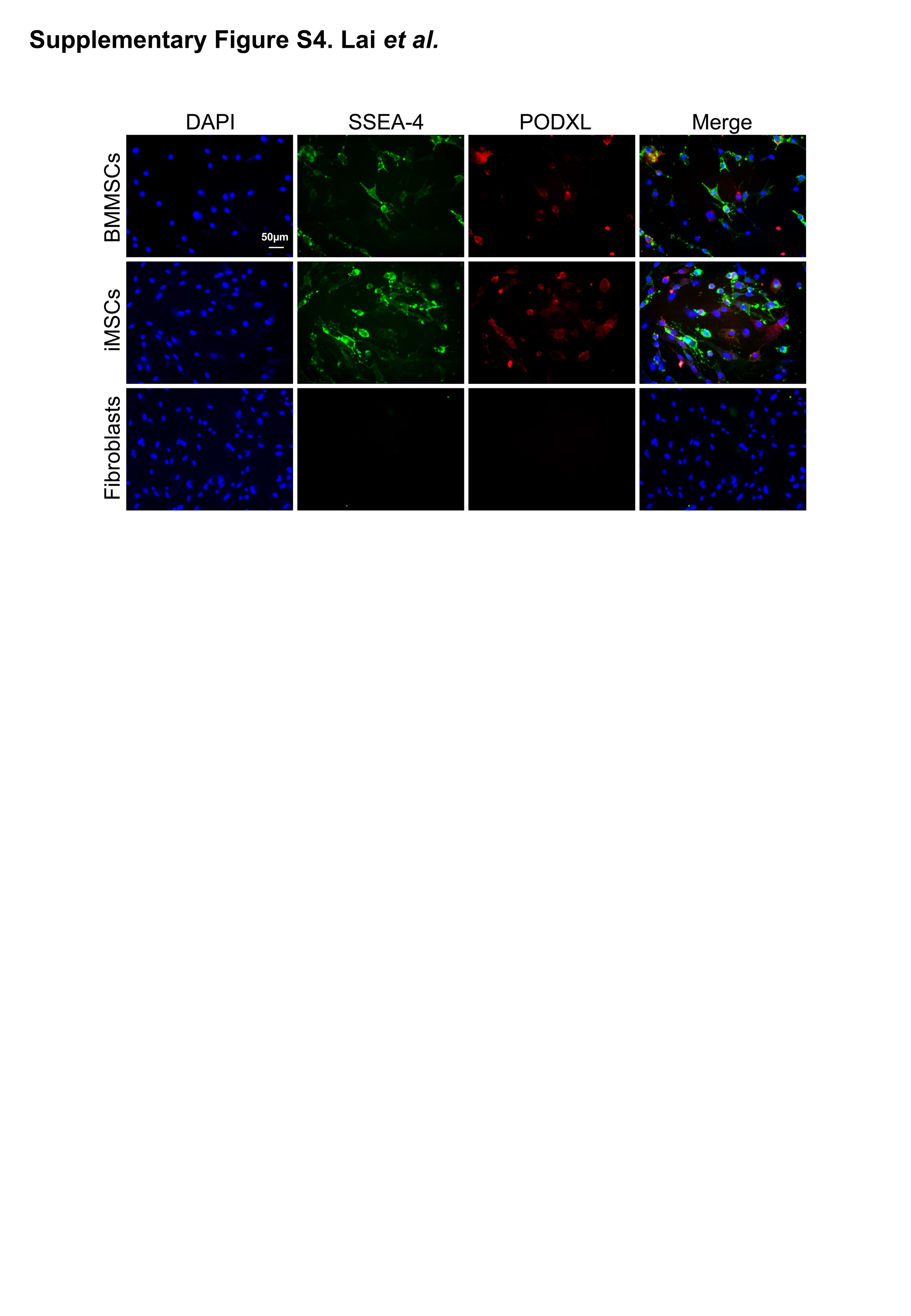
Figure S4. SSEA-4 and PODXL abundantly expressed in iMSCs cultured for 8 passages.**

Dermal fibroblasts were treated with the chemical cocktail (6C+3GF) and sorted with SSEA-4 and PODXL antibodies. Then the iMSCs were cultured in regular MSC medium (DMEM-LG + 10% FBS) for 8 passages. Representative immunofluorescent images of SSEA-4 and PODXL in BMMSCs, iMSCs (at passage 8), and fibroblasts are shown. Scale bar: 50 μm. The nuclei were counterstained with DAPI.


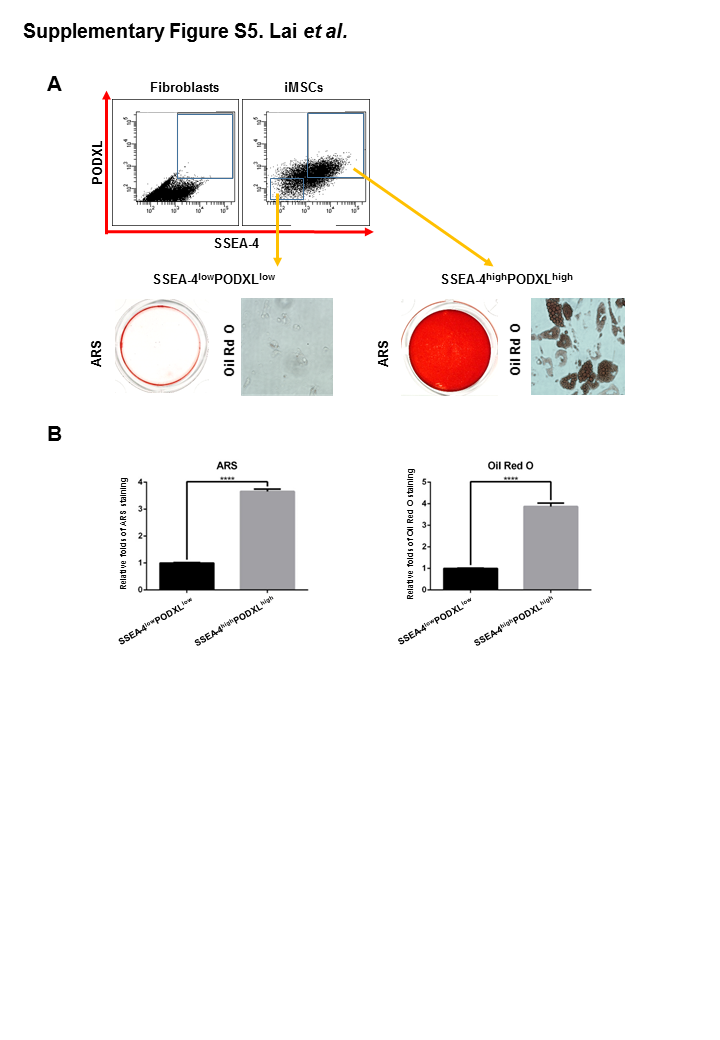
**Figure S5. Only SSEA-4^high^PODXL^high^ cells can differentiate into osteocytes and adipocytes.**
**(A)** iMSCs with SSEA-4^low^PODXL^low^ and SSEA-4^high^PODXL^high^ derived from dermal fibroblasts were cultured in osteogenic and adipogenic induction medium.

**(B)** The quantification results of the Alizarin Red S and the Oil Red O staining (n = 6). *****p*<0.0001.


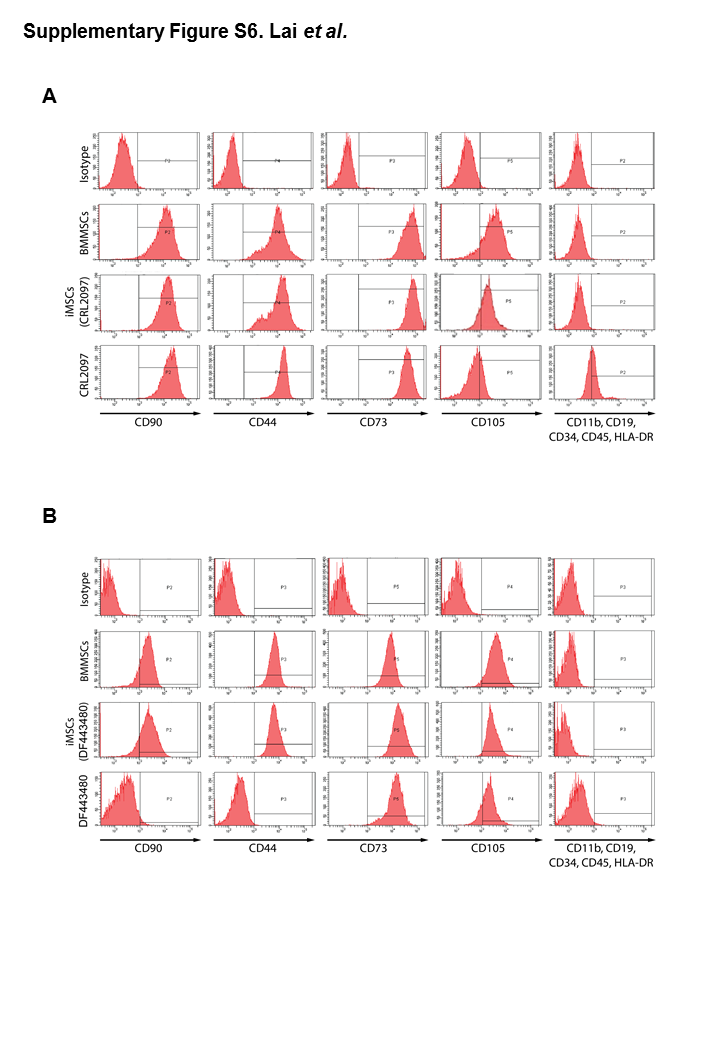
**Figure S6. The expression levels of MSC markers in iMSCs.**

Traditional MSC markers in the ISCT’s proclamation were examined by flow cytometry with the indicated antibody or isotype control. BMMSCs served as the control. iMSCs derived from primary dermal fibroblasts **(A)** CRL2097 and **(B)** DF443480 were examined. Both iMSCs and BMMSCs expressed the MSC traditional markers, CD90, CD44, CD73 and CD105, and did not express CD11b, CD19, CD34, CD45, and HLA-DR (BD Biosciences, San Diego, CA, USA, Stemflow™ hMSC Negative Antibody Cocktail, #562530).

**
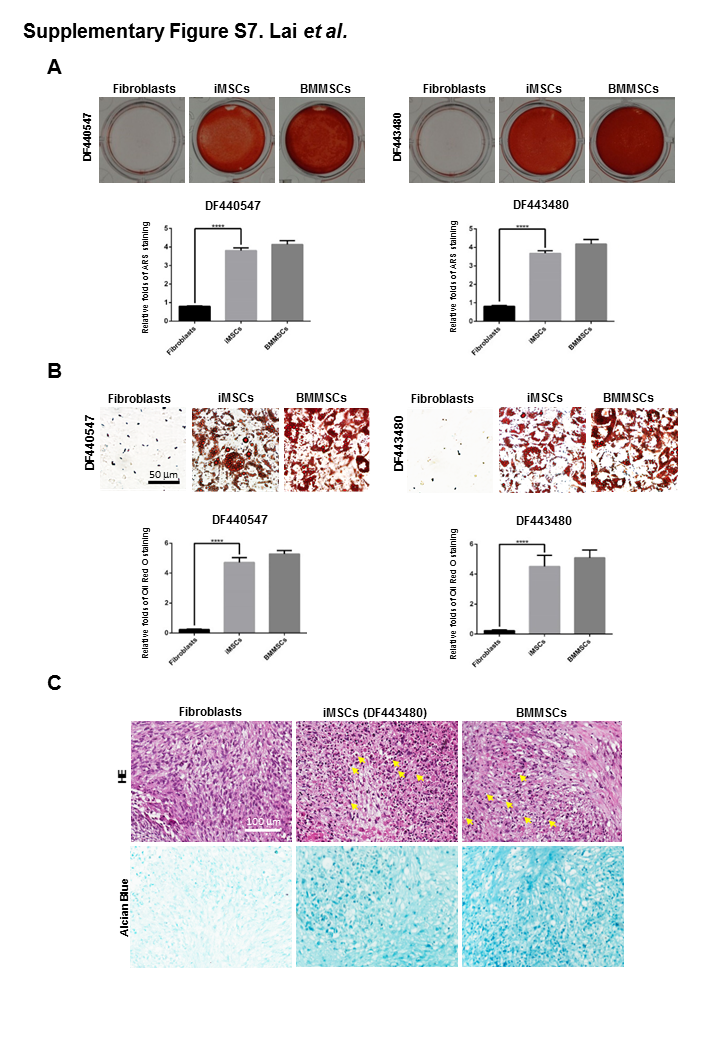
Figure S7. The multipotency of iMSCs induced from fibroblasts of different donors.**

**(A)** The iMSCs derived from human adult abdominal fibroblasts (DF440547 and DF443480) exhibit osteogenesis abilities comparable to those of BMMSCs. Indicated fibroblasts, iMSCs derived from abdominal fibroblasts, and BMMSCs were cultured in osteogenic induction medium for 21 days. Then, they were assayed by Alizarin Red staining (ARS) (upper panel). The ARS signal was quantified by measuring the optical density (O.D.) at 550 nm (lower panel) (n=6). *****p*<0.0001.

**(B)** The iMSCs derived from human adult abdominal fibroblasts (DF440547 and DF443480) exhibit adipogenesis abilities comparable to those of BMMSCs. Indicated fibroblasts, iMSCs, and BMMSCs were cultured in adipocyte induction medium for 21 days, and the lipid drops were then stained with Oil Red O (upper panel). Scale bar: 50 μm. The Oil Red O signal was quantified by measuring the O.D. at 530 nm (lower panel) (n=6). *****p*<0.0001.

**(C)** The iMSCs derived from human abdominal fibroblasts (DF443480) exhibit chondrogenesis abilities comparable to those of BMMSCs. The lacunae structure (revealed by hematoxylin and eosin staining, HE stain, upper panel) (marked by a yellow arrow) and the proteoglycans of cartilage (revealed by Alcian Blue staining, lower panel) were examined to evaluate the capacity of cells to differentiate into chondrocytes at day 21. Three independent experiments were performed. Scale bar: 100 μm.


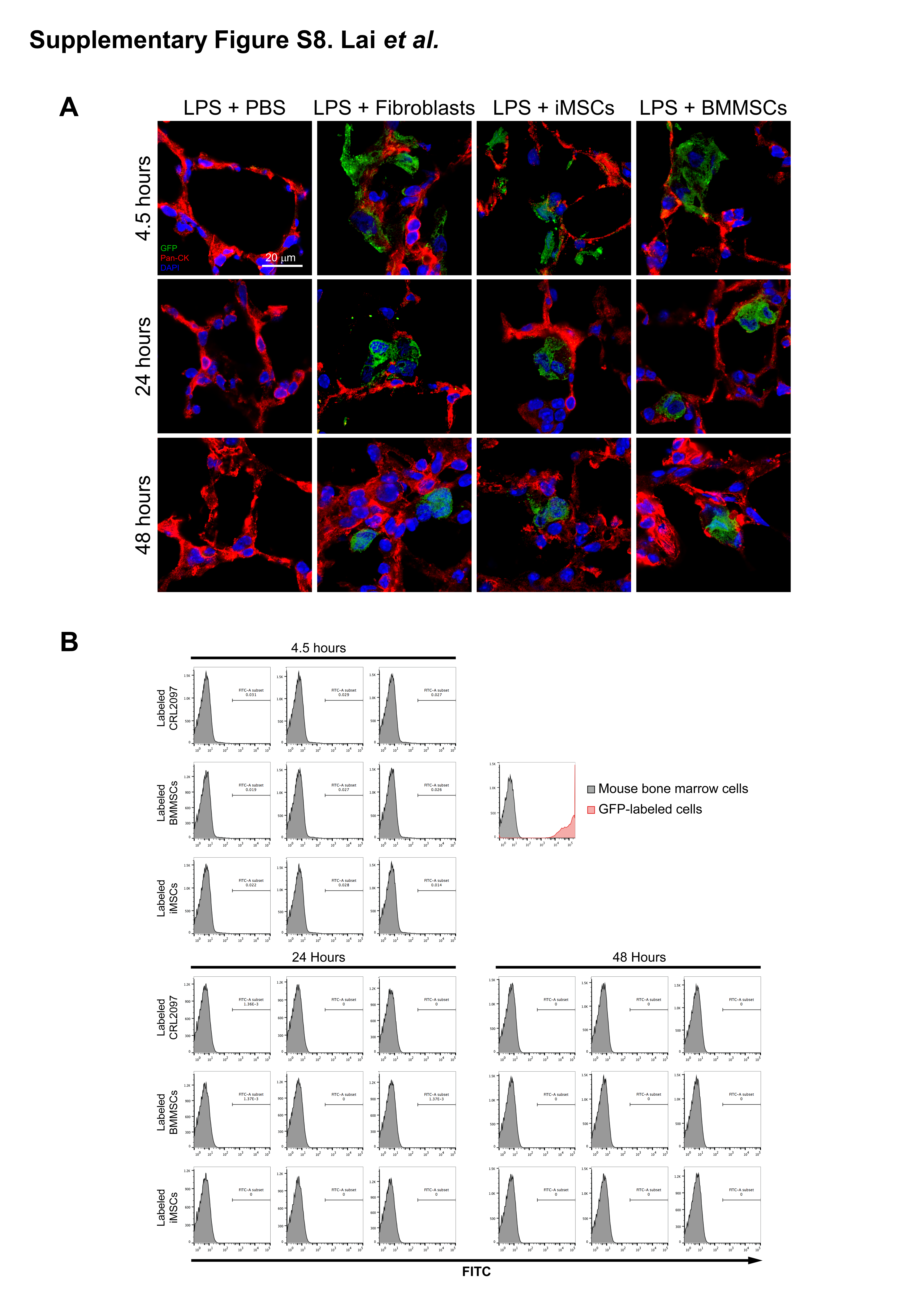
**Figure S8.** **iMSCs and BMMSCs did not contribute to mouse alveolar tissues or migrate to bone marrow within 48-hour period.** 10^6^ of the GFP-labeled human fibroblasts, iMSCs, and BMMSCs were injected into the mice after LPS-injection for 4 hours, and the mice were sacrificed at different time points. **(A)** GFP-labeled human fibroblasts, iMSC, and BMMSCs did not stain with epithelial cell marker pan-cytokeratin (pan-CK) at 4.5-, 24- and 48-hour post-injury. The pan-CK antibody can detect both human and mouse epithelial cells. Scale bar: 20 μm. **(B)** Flow cytometry analysis of GFP-labeled cells in mouse bone marrow after 4.5-, 24-, and 48-hour post-injury. The right upper panel, GFP label human cells were mixed with primary bone marrow cells in vitro and analyzed by flow cytometry. The other panels, the primary bone marrow cells were isolated after the injection of GFP-labelled cells at the indicated post-injury time points. Each cell type has the data from three different mice.


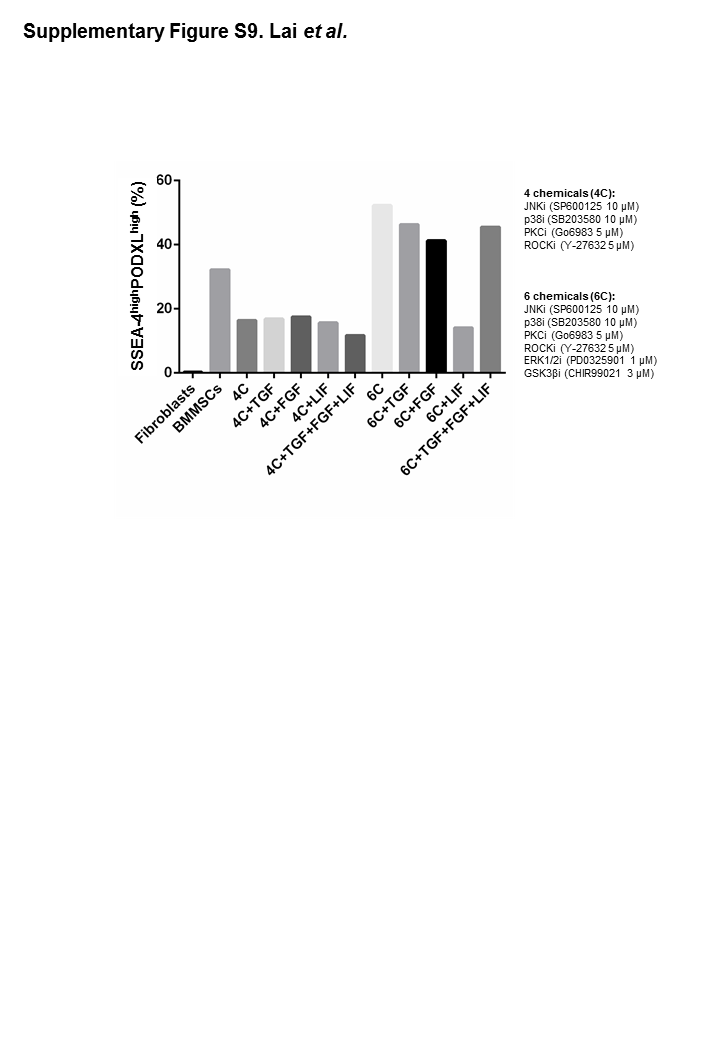


**Figure S9. Cytokines are dispensable for the conversion of human fibroblasts into iMSCs.**

Fibroblasts were treated with four or six chemicals combined with the indicated cytokines and then subjected to flow cytometry analysis at day 6 to quantify iMSC conversion efficiency (SSEA-4^high^PODXL^high^ population). The addition of growth factors did not promote the iMSC conversion of fibroblasts treated with the four or six chemical cocktails.

**Supplemental Materials and Methods**

**Colony forming unit-fibroblast (CFU-F) assay**

The experiment was performed by using cell sorter (FACS AriaTM II, BD Biosciences, San Diego, CA, USA) to plate 500 cells per well of 6-well plates containing culture medium. After 14 days of incubation, the cells were fixed with 100% methanol and then stained with 0.5% crystal violet solution. The number of CFU-F was counted by the definition that a colony has at least 50 cells using an optical inverted microscope (Olympus CK40, Tokyo, Japan).

**Immunofluorescence assay**

Cells were incubated with FITC-conjugated anti-human SSEA-4 (clone MC-813-70; eBioscience, San Diego, CA, USA) or PE-conjugated anti-human PODXL (clone B34D1.3; eBioscience) for 1 hour at room temperature and then washed 3 times with PBS. The nuclei of cells were counterstained with 4',6-diamidino-2-phenylindole (DAPI) after fixation, and their fluorescent images were acquired using a LAS-4000 image system (Fujifilm, Tokyo, Japan). Finally, the brightness and contrast of the whole images were adjusted linearly using Multi Gauge version 3.0 (Fujifilm).

**Lentivirus production and cells infection**

The 293T cells were seeded with 10^6^ cells (per well of 6-well plates) for generation of lentivirus. After 24 hours, the 293T cells were transfected with 1 μg of the pLKO_AS3w.eGFP.bsd, 0.9 μg of pCMVR8.91, and 0.1 μg of pMD.G (National RNAi Core Facility, Taipei, Taiwan) via the Turbofect transfection reagent (Thermo Fisher Scientific, Waltham, MA, USA). At 24 hours after transfection, the medium was changed to HG-DMEM with 10% FBS and 1% BSA harvest medium. At 48 hours or 72 hours, the supernatants were harvested. For infection, 10^6^ fibroblasts, iMSCs, or BMMSCs were seeded (per well of 6-well plates) and later incubated with lentivirus (multiplicity of infection = 10) for one day. The cells were selected with 10 μg/ml blasticidin (Thermo Fisher Scientific) for another day. The GFP-labeled cells were then isolated using a cell sorter (FACS Aria^TM^ II, BD Biosciences) and cultured in medium containing 10 μg/ml blasticidin.

**Immunofluorescence analysis of lung**

After cervical dislocation, lungs were inflated with Tissue-Tek O.C.T^TM^ Compound and Cryomolds (Sakura, Alphen aan den Rijn, Netherlands) and flash frozen at -80℃. Sections (5 μm) were prepared by pathology core (Pathology core, IBMS, Taipei, Taiwan) and stored at -80℃. Sections were fixed in 4% paraformaldehyde (PFA) at room temperature for 5 minutes, permeabilized with 0.1% Triton X-100 at room temperature for 5 minutes, and blocked by 5% FBS in PBS for 30 minutes at room temperature. Slides were incubated with primary antibodies with the following conditions, mouse anti-pan-cytokeratin (1:200; Abcam, #ab27988, Cambridge, UK) at 4℃ overnight, chicken anti-GFP (1:500; Abcam, #ab13970) at room temperature 30 minutes. Slides were washed 3 times with PBS, then incubated with secondary antibodies with 1:200 dilutions at room temperature for 30 minutes. Then the slides were stained with 1μM DAPI for 5 minutes and mounted by Prolong Gold (Thermo Fisher Scientific).

**Table S1. Chemical compounds tested for inducing iMSCs from fibroblasts.**

| **Name** | **Mechanism** | **Source** | **Concentration** | **Structure** |
| --- | --- | --- | --- | --- |
| RepSox | TGF-β inhibitor | TOCRIS | 10 μM | 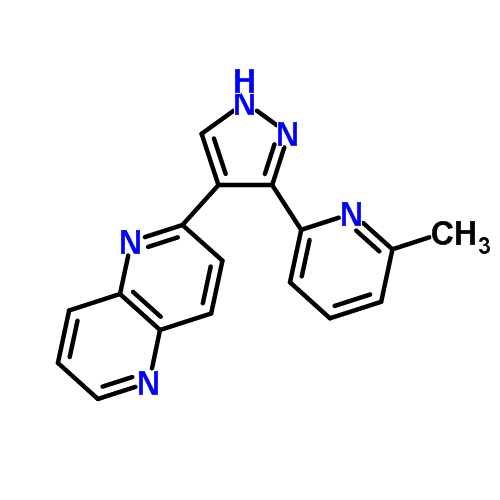 |
| CHIR99021 | GSK3β inhibitor | TOCRIS | 3 μM | 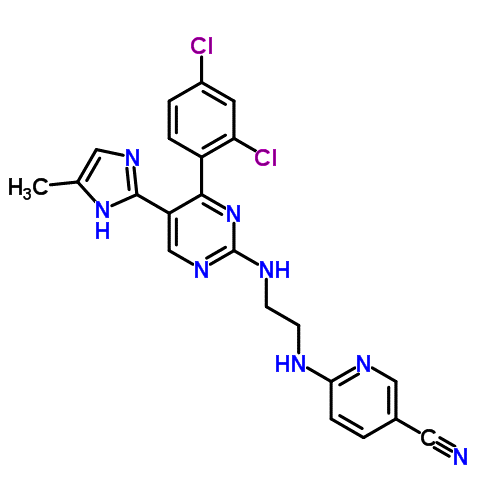 |
| Tranylcypromine | H3K4 demethylation inhibitor | TOCRIS | 10 μM | 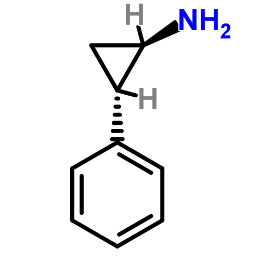 |
| 3-Deazaneplanocin A,  DZNep, | S-adenosylmethionine-dependent methyltransferase | TOCRIS | 0.1 μM | **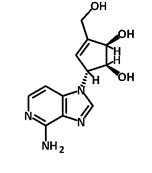** |
| TTNPB | potent analog of retinoic acid | TOCRIS | 1 μM | 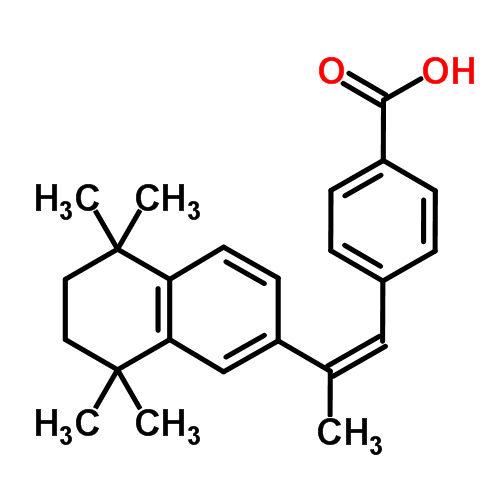 |
| SP600125 | JNK inhibitor | LC Laboratories | 10 μM | 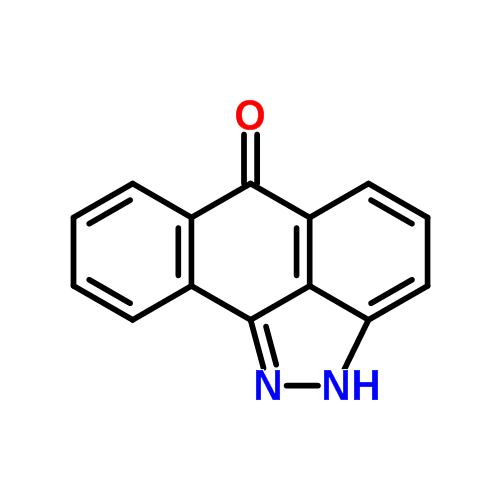 |
| SB202190 | p38 inhibitor | LC Laboratories | 10 μM | 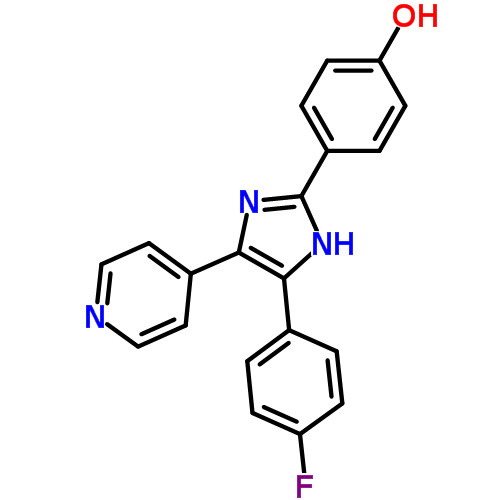 |
| SB203580 | p38 inhibitor | LC Laboratories | 10 μM | 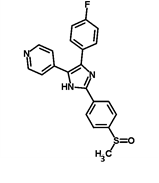 |
| PD0325901 | Erk inhibitor | LC Laboratories | 1 μM | 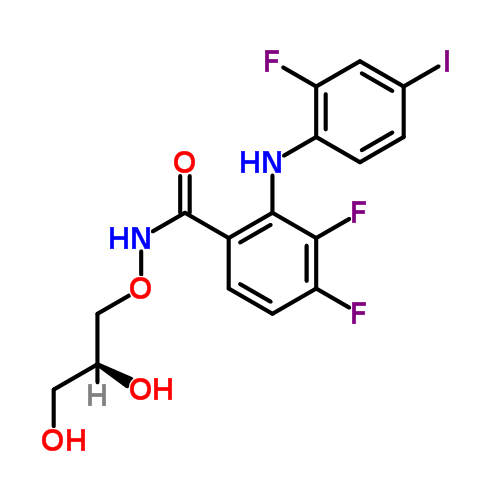 |
| Go 6983 | PKC inhibitor | TOCRIS | 5 μM | 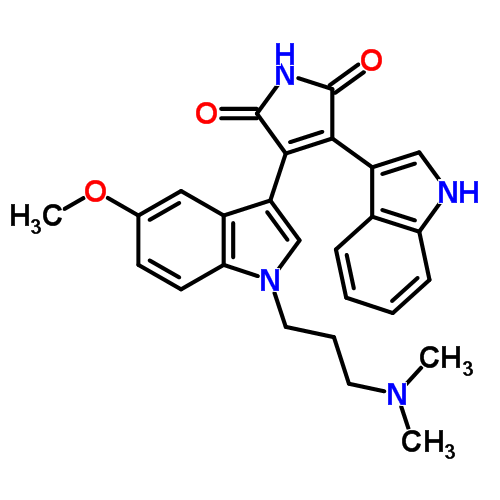 |
| Y-27632 | ROCK inhibitor | LC Laboratories | 5 µM | 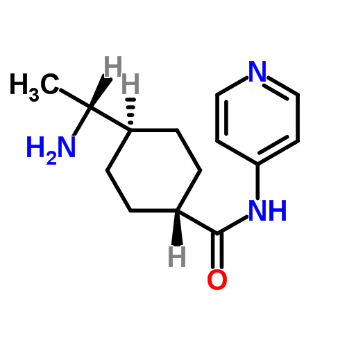 |
| LiCl | GSK-3β inhibitor, LSD1 inhibitor | Sigma | 10 mM | 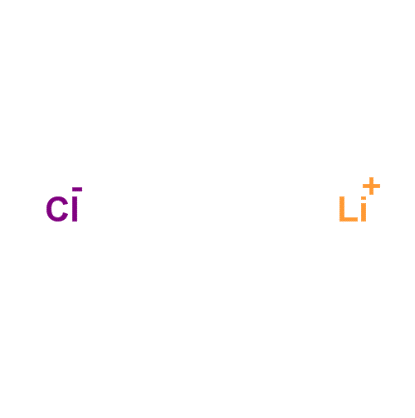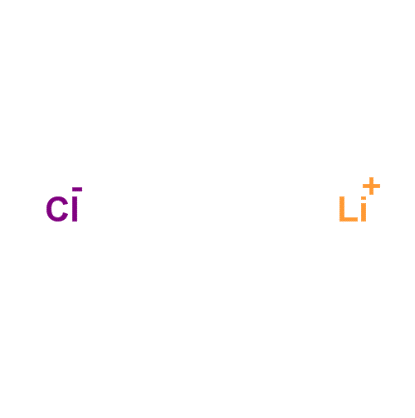 |
| Rapamycin | mTOR inhibitor | TOCRIS | 0.3 nM | 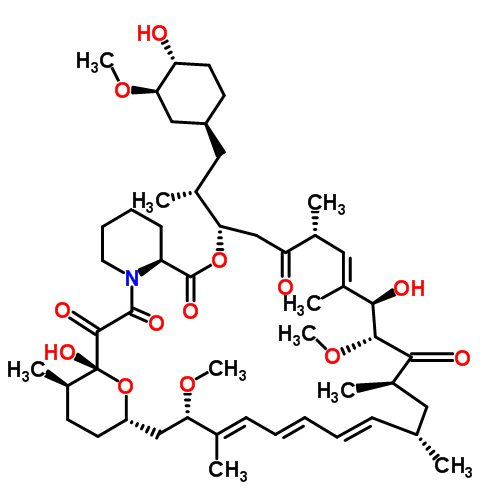 |
| Quercetin | HIF1α activator | TOCRIS | 1 μM | 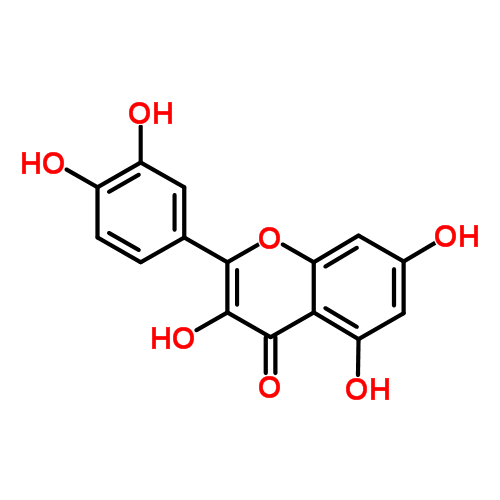 |
| CoCl_2_ | HIF1α activator | Sigma | 100 μM | 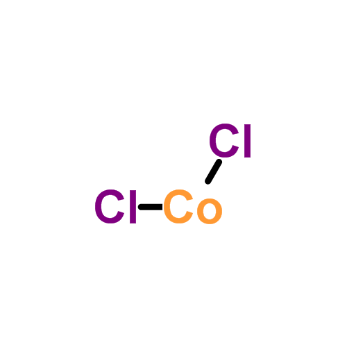 |
| ML228 | HIF1α activator | TOCRIS | 10 μM | 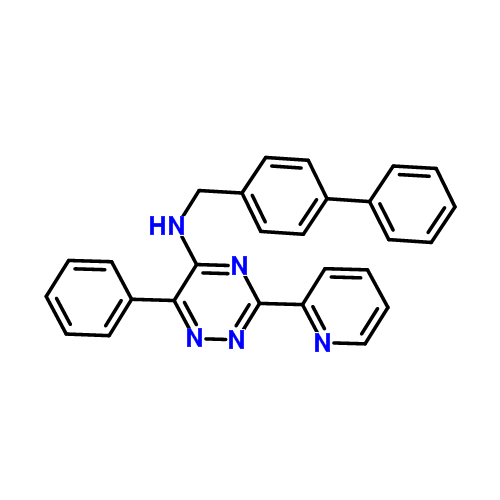 |
| Sodium butyrate | HDAC inhibitor | Sigma | 1 mM | 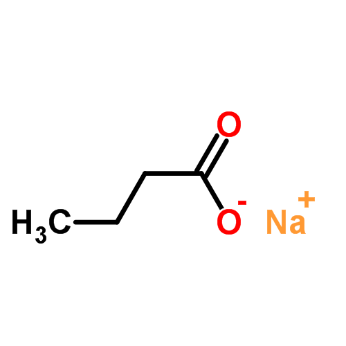 |
| SB431542 | ALK4, ALK5, and ALK7 inhibitor | Sigma | 10 μM | 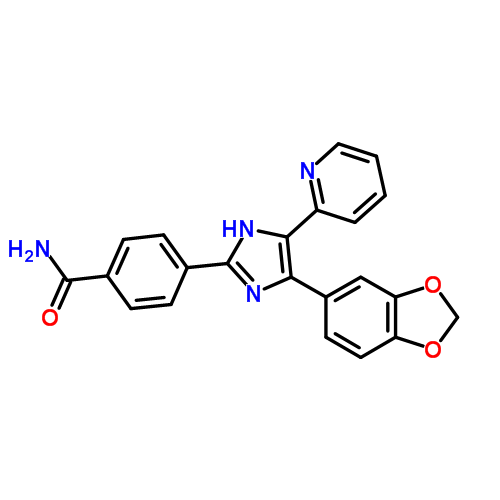 |
| 5-aza-CR,AZA | DMNT inhibitor | Sigma | 0.5 mM | 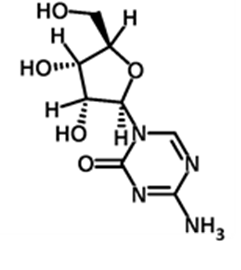 |
| I-BET 151 | BET family bromodomain inhibitor | TOCRIS | 2 μM | 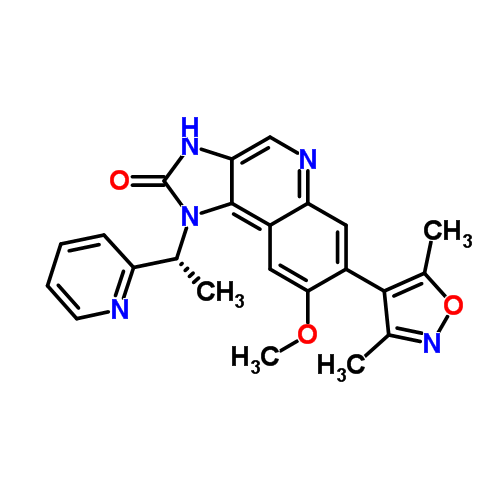 |
| A83-01 | TGF-β signaling inhibitor | Sigma | 2 μM | 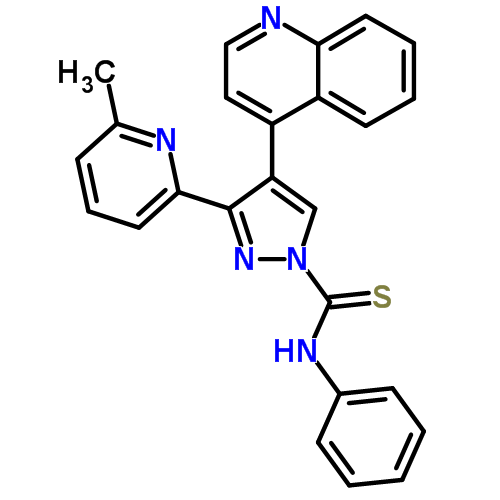 |
| N-Acetylcysteine, NAC | anti-oxidant small molecule | Sigma | 2 mM | 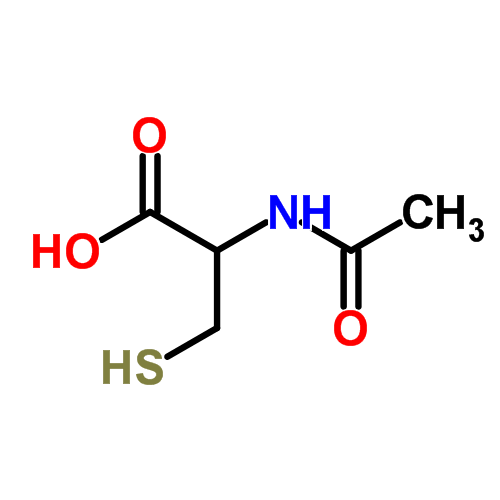 |
| Glutathione,  GSH | Apoptosis regulator as a substrate of ROS scavenging enzymes | Sigma | 2 mM | 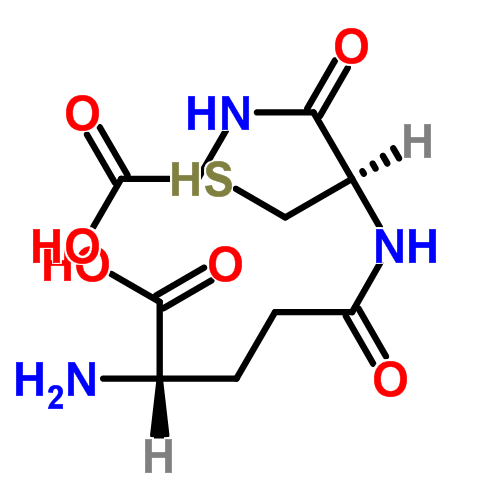 |
| Vitamin C, VitC | Nanog enhancer, JAK/STAT activator | Sigma | 50 ng/mL | 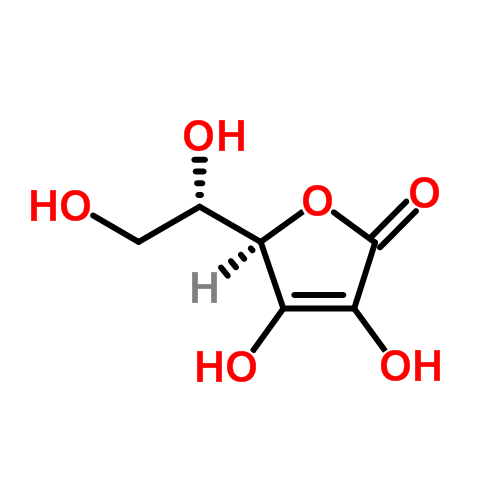 |
| Thiazovivin | Rho-associated protein kinase  inhibitor | LC laboratories | 1 μM | 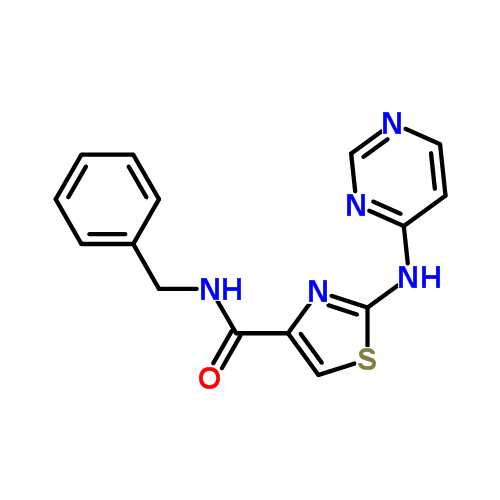 |
| Rolipram | cAMP agonist | LC laboratories | 10 μM | 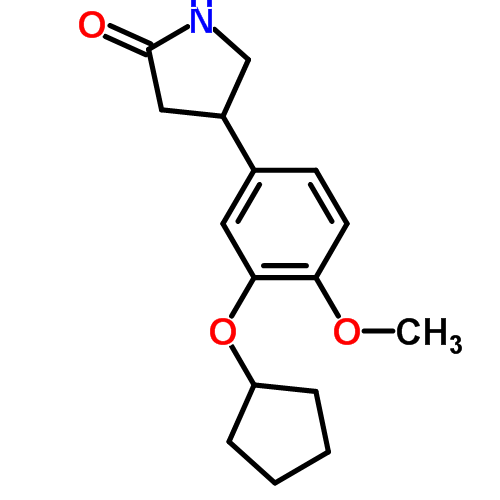 |
| Dasatinib | Replace Sox2, Src family tyrosine kinase  inhibitor | LC laboratories | 10 μM | 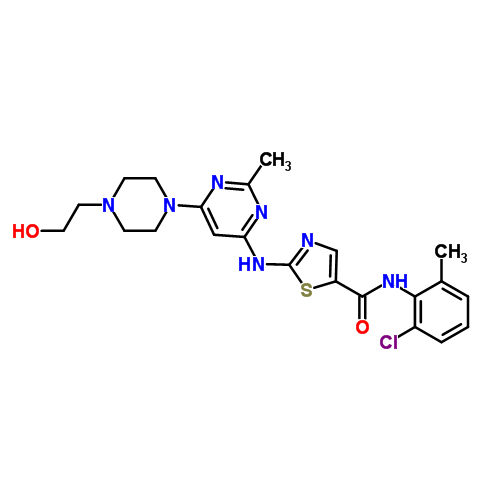 |
| PP1 | Replace Sox2, Src family tyrosine kinase  inhibitor | LC laboratories | 10 μM | 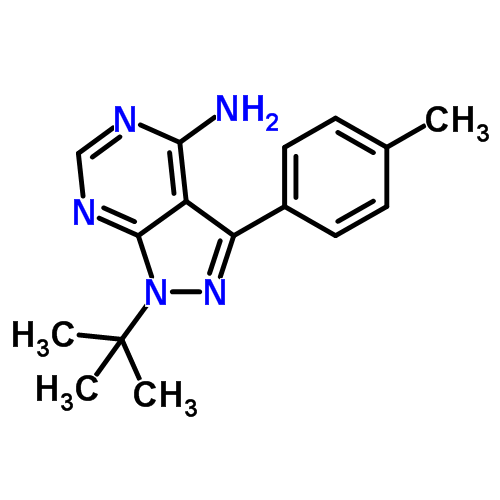 |
| LY294002 | Inhibitor of PI3K/AKT | LC laboratories | 20 μM | 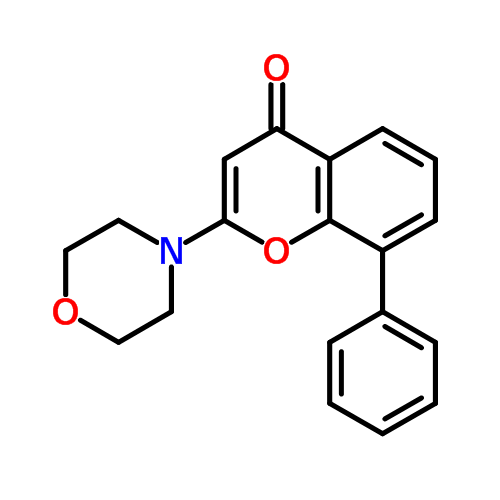 |
| Fasudil | Rho kinase inhibitor | LC laboratories | 0.2 μM | 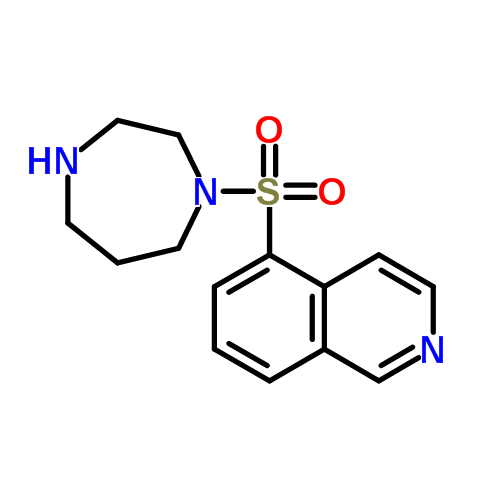 |

**Table S2. 418 probes for principle component anaylsis (PCA)**

| PROBEID | ENTREZID | SYMBOL | GENENAME | GO | TERM |
| --- | --- | --- | --- | --- | --- |
| 1552621_at | 5439 | POLR2J | polymerase (RNA) II (DNA directed) polypeptide J, 13.3kDa | GO:0035019 | somatic stem cell maintenance |
| 1552622_s_at | 5439 | POLR2J | polymerase (RNA) II (DNA directed) polypeptide J, 13.3kDa | GO:0035019 | somatic stem cell maintenance |
| 1552973_at | 7484 | WNT9B | wingless-type MMTV integration site family, member 9B | GO:0072038 | mesenchymal stem cell maintenance involved in nephron morphogenesis |
| 1552982_a_at | 2249 | FGF4 | fibroblast growth factor 4 | GO:0019827 | stem cell maintenance |
| 1553874_a_at | 84891 | ZSCAN10 | zinc finger and SCAN domain containing 10 | GO:0035019 | somatic stem cell maintenance |
| 1553875_s_at | 84891 | ZSCAN10 | zinc finger and SCAN domain containing 10 | GO:0035019 | somatic stem cell maintenance |
| 1554052_at | 23019 | CNOT1 | CCR4-NOT transcription complex, subunit 1 | GO:2000036 | regulation of stem cell maintenance |
| 1554411_at | 1499 | CTNNB1 | catenin (cadherin-associated protein), beta 1, 88kDa | GO:0019827 | stem cell maintenance |
| 1555677_s_at | 8243 | SMC1A | structural maintenance of chromosomes 1A | GO:0019827 | stem cell maintenance |
| 1555836_at | 5431 | POLR2B | polymerase (RNA) II (DNA directed) polypeptide B, 140kDa | GO:0035019 | somatic stem cell maintenance |
| 1555837_s_at | 5431 | POLR2B | polymerase (RNA) II (DNA directed) polypeptide B, 140kDa | GO:0035019 | somatic stem cell maintenance |
| 1556156_at | 2103 | ESRRB | estrogen-related receptor beta | GO:0019827 | stem cell maintenance |
| 1556925_at | 9126 | SMC3 | structural maintenance of chromosomes 3 | GO:0019827 | stem cell maintenance |
| 1557675_at | 5894 | RAF1 | Raf-1 proto-oncogene, serine/threonine kinase | GO:0035019 | somatic stem cell maintenance |
| 1557706_at | 22882 | ZHX2 | zinc fingers and homeoboxes 2 | GO:0035019 | somatic stem cell maintenance |
| 1557767_at | 339345 | NANOS2 | nanos homolog 2 (Drosophila) | GO:0030718 | germ-line stem cell maintenance |
| 1558315_s_at | 84376 | HOOK3 | hook microtubule-tethering protein 3 | GO:0097150 | neuronal stem cell maintenance |
| 1558682_at | 8091 | HMGA2 | high mobility group AT-hook 2 | GO:2000036 | regulation of stem cell maintenance |
| 1558683_a_at | 8091 | HMGA2 | high mobility group AT-hook 2 | GO:2000036 | regulation of stem cell maintenance |
| 1559142_at | 7994 | KAT6A | K(lysine) acetyltransferase 6A | GO:0035019 | somatic stem cell maintenance |
| 1559891_at | 8091 | HMGA2 | high mobility group AT-hook 2 | GO:2000036 | regulation of stem cell maintenance |
| 1561633_at | 8091 | HMGA2 | high mobility group AT-hook 2 | GO:2000036 | regulation of stem cell maintenance |
| 1561651_s_at | 6886 | TAL1 | T-cell acute lymphocytic leukemia 1 | GO:2000036 | regulation of stem cell maintenance |
| 1565702_at | 4089 | SMAD4 | SMAD family member 4 | GO:0035019 | somatic stem cell maintenance |
| 1565703_at | 4089 | SMAD4 | SMAD family member 4 | GO:0035019 | somatic stem cell maintenance |
| 1567223_at | 8091 | HMGA2 | high mobility group AT-hook 2 | GO:2000036 | regulation of stem cell maintenance |
| 1567224_at | 8091 | HMGA2 | high mobility group AT-hook 2 | GO:2000036 | regulation of stem cell maintenance |
| 1567906_at | 6659 | SOX4 | SRY (sex determining region Y)-box 4 | GO:0035019 | somatic stem cell maintenance |
| 1568286_at | 8091 | HMGA2 | high mobility group AT-hook 2 | GO:2000036 | regulation of stem cell maintenance |
| 1568287_at | 8091 | HMGA2 | high mobility group AT-hook 2 | GO:2000036 | regulation of stem cell maintenance |
| 1569073_x_at | 6597 | SMARCA4 | SWI/SNF related, matrix associated, actin dependent regulator of chromatin, subfamily a, member 4 | GO:0019827 | stem cell maintenance |
| 200860_s_at | 23019 | CNOT1 | CCR4-NOT transcription complex, subunit 1 | GO:2000036 | regulation of stem cell maintenance |
| 200861_at | 23019 | CNOT1 | CCR4-NOT transcription complex, subunit 1 | GO:2000036 | regulation of stem cell maintenance |
| 200878_at | 2034 | EPAS1 | endothelial PAS domain protein 1 | GO:0035019 | somatic stem cell maintenance |
| 200879_s_at | 2034 | EPAS1 | endothelial PAS domain protein 1 | GO:0035019 | somatic stem cell maintenance |
| 201244_s_at | 5894 | RAF1 | Raf-1 proto-oncogene, serine/threonine kinase | GO:0035019 | somatic stem cell maintenance |
| 201367_s_at | 678 | ZFP36L2 | ZFP36 ring finger protein-like 2 | GO:0035019 | somatic stem cell maintenance |
| 201368_at | 678 | ZFP36L2 | ZFP36 ring finger protein-like 2 | GO:0035019 | somatic stem cell maintenance |
| 201369_s_at | 678 | ZFP36L2 | ZFP36 ring finger protein-like 2 | GO:0035019 | somatic stem cell maintenance |
| 201416_at | 6659 | SOX4 | SRY (sex determining region Y)-box 4 | GO:0035019 | somatic stem cell maintenance |
| 201417_at | 6659 | SOX4 | SRY (sex determining region Y)-box 4 | GO:0035019 | somatic stem cell maintenance |
| 201418_s_at | 6659 | SOX4 | SRY (sex determining region Y)-box 4 | GO:0035019 | somatic stem cell maintenance |
| 201423_s_at | 8451 | CUL4A | cullin 4A | GO:0035019 | somatic stem cell maintenance |
| 201424_s_at | 8451 | CUL4A | cullin 4A | GO:0035019 | somatic stem cell maintenance |
| 201533_at | 1499 | CTNNB1 | catenin (cadherin-associated protein), beta 1, 88kDa | GO:0019827 | stem cell maintenance |
| 201589_at | 8243 | SMC1A | structural maintenance of chromosomes 1A | GO:0019827 | stem cell maintenance |
| 201679_at | 51593 | SRRT | serrate, RNA effector molecule | GO:0097150 | neuronal stem cell maintenance |
| 201680_x_at | 51593 | SRRT | serrate, RNA effector molecule | GO:0097150 | neuronal stem cell maintenance |
| 201726_at | 1994 | ELAVL1 | ELAV like RNA binding protein 1 | GO:2000036 | regulation of stem cell maintenance |
| 201727_s_at | 1994 | ELAVL1 | ELAV like RNA binding protein 1 | GO:2000036 | regulation of stem cell maintenance |
| 201803_at | 5431 | POLR2B | polymerase (RNA) II (DNA directed) polypeptide B, 140kDa | GO:0035019 | somatic stem cell maintenance |
| 202035_s_at | 6422 | SFRP1 | secreted frizzled-related protein 1 | GO:0035019 | somatic stem cell maintenance |
| 202036_s_at | 6422 | SFRP1 | secreted frizzled-related protein 1 | GO:0035019 | somatic stem cell maintenance |
| 202037_s_at | 6422 | SFRP1 | secreted frizzled-related protein 1 | GO:0035019 | somatic stem cell maintenance |
| 202060_at | 9646 | CTR9 | CTR9, Paf1/RNA polymerase II complex component | GO:0019827 | stem cell maintenance |
| 202093_s_at | 54623 | PAF1 | Paf1, RNA polymerase II associated factor, homolog (S. cerevisiae) | GO:0019827 | stem cell maintenance |
| 202160_at | 1387 | CREBBP | CREB binding protein | GO:0030718 | germ-line stem cell maintenance |
| 202174_s_at | 5108 | PCM1 | pericentriolar material 1 | GO:0097150 | neuronal stem cell maintenance |
| 202261_at | 6944 | VPS72 | vacuolar protein sorting 72 homolog (S. cerevisiae) | GO:0035019 | somatic stem cell maintenance |
| 202306_at | 5436 | POLR2G | polymerase (RNA) II (DNA directed) polypeptide G | GO:0035019 | somatic stem cell maintenance |
| 202393_s_at | 7071 | KLF10 | Kruppel-like factor 10 | GO:0035019 | somatic stem cell maintenance |
| 202423_at | 7994 | KAT6A | K(lysine) acetyltransferase 6A | GO:0035019 | somatic stem cell maintenance |
| 202443_x_at | 4853 | NOTCH2 | notch 2 | GO:0019827 | stem cell maintenance |
| 202445_s_at | 4853 | NOTCH2 | notch 2 | GO:0019827 | stem cell maintenance |
| 202526_at | 4089 | SMAD4 | SMAD family member 4 | GO:0035019 | somatic stem cell maintenance |
| 202527_s_at | 4089 | SMAD4 | SMAD family member 4 | GO:0035019 | somatic stem cell maintenance |
| 202586_at | 5441 | POLR2L | polymerase (RNA) II (DNA directed) polypeptide L, 7.6kDa | GO:0035019 | somatic stem cell maintenance |
| 202610_s_at | 9282 | MED14 | mediator complex subunit 14 | GO:0019827 | stem cell maintenance |
| 202611_s_at | 9282 | MED14 | mediator complex subunit 14 | GO:0019827 | stem cell maintenance |
| 202612_s_at | 9282 | MED14 | mediator complex subunit 14 | GO:0019827 | stem cell maintenance |
| 202634_at | 5440 | POLR2K | polymerase (RNA) II (DNA directed) polypeptide K, 7.0kDa | GO:0035019 | somatic stem cell maintenance |
| 202635_s_at | 5440 | POLR2K | polymerase (RNA) II (DNA directed) polypeptide K, 7.0kDa | GO:0035019 | somatic stem cell maintenance |
| 202723_s_at | 2308 | FOXO1 | forkhead box O1 | GO:0097150 | neuronal stem cell maintenance |
| 202724_s_at | 2308 | FOXO1 | forkhead box O1 | GO:0097150 | neuronal stem cell maintenance |
| 202725_at | 5430 | POLR2A | polymerase (RNA) II (DNA directed) polypeptide A, 220kDa | GO:0035019 | somatic stem cell maintenance |
| 202816_s_at | 6760 | SS18 | synovial sarcoma translocation, chromosome 18 | GO:0097150 | neuronal stem cell maintenance |
| 202817_s_at | 6760 | SS18 | synovial sarcoma translocation, chromosome 18 | GO:0097150 | neuronal stem cell maintenance |
| 202935_s_at | 6662 | SOX9 | SRY (sex determining region Y)-box 9 | GO:0035019 | somatic stem cell maintenance |
| 202936_s_at | 6662 | SOX9 | SRY (sex determining region Y)-box 9 | GO:0035019 | somatic stem cell maintenance |
| 203075_at | 4087 | SMAD2 | SMAD family member 2 | GO:0035019 | somatic stem cell maintenance |
| 203076_s_at | 4087 | SMAD2 | SMAD family member 2 | GO:0035019 | somatic stem cell maintenance |
| 203077_s_at | 4087 | SMAD2 | SMAD family member 2 | GO:0035019 | somatic stem cell maintenance |
| 203239_s_at | 4849 | CNOT3 | CCR4-NOT transcription complex, subunit 3 | GO:2000036 | regulation of stem cell maintenance |
| 203345_s_at | 22823 | MTF2 | metal response element binding transcription factor 2 | GO:0019827 | stem cell maintenance |
| 203346_s_at | 22823 | MTF2 | metal response element binding transcription factor 2 | GO:0019827 | stem cell maintenance |
| 203347_s_at | 22823 | MTF2 | metal response element binding transcription factor 2 | GO:0019827 | stem cell maintenance |
| 203393_at | 3280 | HES1 | hes family bHLH transcription factor 1 | GO:0035019 | somatic stem cell maintenance |
| 203394_s_at | 3280 | HES1 | hes family bHLH transcription factor 1 | GO:0035019 | somatic stem cell maintenance |
| 203395_s_at | 3280 | HES1 | hes family bHLH transcription factor 1 | GO:0035019 | somatic stem cell maintenance |
| 203440_at | 1000 | CDH2 | cadherin 2, type 1, N-cadherin (neuronal) | GO:0097150 | neuronal stem cell maintenance |
| 203441_s_at | 1000 | CDH2 | cadherin 2, type 1, N-cadherin (neuronal) | GO:0097150 | neuronal stem cell maintenance |
| 203451_at | 8861 | LDB1 | LIM domain binding 1 | GO:0035019 | somatic stem cell maintenance |
| 203506_s_at | 9968 | MED12 | mediator complex subunit 12 | GO:0019827 | stem cell maintenance |
| 203525_s_at | 324 | APC | adenomatous polyposis coli | GO:0035019 | somatic stem cell maintenance |
| 203526_s_at | 324 | APC | adenomatous polyposis coli | GO:0035019 | somatic stem cell maintenance |
| 203527_s_at | 324 | APC | adenomatous polyposis coli | GO:0035019 | somatic stem cell maintenance |
| 203556_at | 22882 | ZHX2 | zinc fingers and homeoboxes 2 | GO:0035019 | somatic stem cell maintenance |
| 203649_s_at | 5320 | PLA2G2A | phospholipase A2, group IIA (platelets, synovial fluid) | GO:0035019 | somatic stem cell maintenance |
| 203664_s_at | 5433 | POLR2D | polymerase (RNA) II (DNA directed) polypeptide D | GO:0035019 | somatic stem cell maintenance |
| 203705_s_at | 8324 | FZD7 | frizzled class receptor 7 | GO:0019827 | stem cell maintenance |
| 203706_s_at | 8324 | FZD7 | frizzled class receptor 7 | GO:0019827 | stem cell maintenance |
| 204107_at | 4800 | NFYA | nuclear transcription factor Y, alpha | GO:2000036 | regulation of stem cell maintenance |
| 204108_at | 4800 | NFYA | nuclear transcription factor Y, alpha | GO:2000036 | regulation of stem cell maintenance |
| 204109_s_at | 4800 | NFYA | nuclear transcription factor Y, alpha | GO:2000036 | regulation of stem cell maintenance |
| 204129_at | 607 | BCL9 | B-cell CLL/lymphoma 9 | GO:0035019 | somatic stem cell maintenance |
| 204131_s_at | 2309 | FOXO3 | forkhead box O3 | GO:0097150 | neuronal stem cell maintenance |
| 204132_s_at | 2309 | FOXO3 | forkhead box O3 | GO:0097150 | neuronal stem cell maintenance |
| 204270_at | 6497 | SKI | SKI proto-oncogene | GO:0035019 | somatic stem cell maintenance |
| 204349_at | 9443 | MED7 | mediator complex subunit 7 | GO:0019827 | stem cell maintenance |
| 204350_s_at | 9443 | MED7 | mediator complex subunit 7 | GO:0019827 | stem cell maintenance |
| 204379_s_at | 2261 | FGFR3 | fibroblast growth factor receptor 3 | GO:0035019 | somatic stem cell maintenance |
| 204380_s_at | 2261 | FGFR3 | fibroblast growth factor receptor 3 | GO:0035019 | somatic stem cell maintenance |
| 204421_s_at | 2247 | FGF2 | fibroblast growth factor 2 (basic) | GO:0035019 | somatic stem cell maintenance |
| 204422_s_at | 2247 | FGF2 | fibroblast growth factor 2 (basic) | GO:0035019 | somatic stem cell maintenance |
| 204832_s_at | 657 | BMPR1A | bone morphogenetic protein receptor, type IA | GO:0019827 | stem cell maintenance |
| 205051_s_at | 3815 | KIT | v-kit Hardy-Zuckerman 4 feline sarcoma viral oncogene homolog | GO:0019827 | stem cell maintenance |
| 205253_at | 5087 | PBX1 | pre-B-cell leukemia homeobox 1 | GO:0035019 | somatic stem cell maintenance |
| 205266_at | 3976 | LIF | leukemia inhibitory factor | GO:0019827 | stem cell maintenance |
| 205286_at | 7022 | TFAP2C | transcription factor AP-2 gamma (activating enhancer binding protein 2 gamma) | GO:0030718 | germ-line stem cell maintenance |
| 205287_s_at | 7022 | TFAP2C | transcription factor AP-2 gamma (activating enhancer binding protein 2 gamma) | GO:0030718 | germ-line stem cell maintenance |
| 205312_at | 6688 | SPI1 | Spi-1 proto-oncogene | GO:0035019 | somatic stem cell maintenance |
| 205977_s_at | 2041 | EPHA1 | EPH receptor A1 | GO:0035019 | somatic stem cell maintenance |
| 206044_s_at | 673 | BRAF | B-Raf proto-oncogene, serine/threonine kinase | GO:0035019 | somatic stem cell maintenance |
| 206061_s_at | 23405 | DICER1 | dicer 1, ribonuclease type III | GO:0019827 | stem cell maintenance |
| 206235_at | 3981 | LIG4 | ligase IV, DNA, ATP-dependent | GO:0035019 | somatic stem cell maintenance |
| 206283_s_at | 6886 | TAL1 | T-cell acute lymphocytic leukemia 1 | GO:2000036 | regulation of stem cell maintenance |
| 206286_s_at | 6997 | TDGF1 | teratocarcinoma-derived growth factor 1 | GO:0035019 | somatic stem cell maintenance |
| 206387_at | 1045 | CDX2 | caudal type homeobox 2 | GO:0035019 | somatic stem cell maintenance |
| 206481_s_at | 9079 | LDB2 | LIM domain binding 2 | GO:0035019 | somatic stem cell maintenance |
| 206510_at | 10736 | SIX2 | SIX homeobox 2 | GO:0072038 | mesenchymal stem cell maintenance involved in nephron morphogenesis |
| 206511_s_at | 10736 | SIX2 | SIX homeobox 2 | GO:0072038 | mesenchymal stem cell maintenance involved in nephron morphogenesis |
| 206783_at | 2249 | FGF4 | fibroblast growth factor 4 | GO:0019827 | stem cell maintenance |
| 207108_s_at | 25836 | NIPBL | Nipped-B homolog (Drosophila) | GO:0019827 | stem cell maintenance |
| 207197_at | 7547 | ZIC3 | Zic family member 3 | GO:0035019 | somatic stem cell maintenance |
| 207401_at | 5629 | PROX1 | prospero homeobox 1 | GO:0097150 | neuronal stem cell maintenance |
| 207443_at | 7101 | NR2E1 | nuclear receptor subfamily 2, group E, member 1 | GO:0035019 | somatic stem cell maintenance |
| 207607_at | 430 | ASCL2 | achaete-scute family bHLH transcription factor 2 | GO:0035019 | somatic stem cell maintenance |
| 207726_at | 2103 | ESRRB | estrogen-related receptor beta | GO:0019827 | stem cell maintenance |
| 207785_s_at | 3516 | RBPJ | recombination signal binding protein for immunoglobulin kappa J region | GO:0035019 | somatic stem cell maintenance |
| 207954_at | 2624 | GATA2 | GATA binding protein 2 | GO:0035019 | somatic stem cell maintenance |
| 207983_s_at | 10735 | STAG2 | stromal antigen 2 | GO:0019827 | stem cell maintenance |
| 208025_s_at | 8091 | HMGA2 | high mobility group AT-hook 2 | GO:2000036 | regulation of stem cell maintenance |
| 208286_x_at | 5460 | POU5F1 | POU class 5 homeobox 1 | GO:0035019 | somatic stem cell maintenance |
| 208387_s_at | 10893 | MMP24 | matrix metallopeptidase 24 (membrane-inserted) | GO:0097150 | neuronal stem cell maintenance |
| 208500_x_at | 27022 | FOXD3 | forkhead box D3 | GO:0035019 | somatic stem cell maintenance |
| 208793_x_at | 6597 | SMARCA4 | SWI/SNF related, matrix associated, actin dependent regulator of chromatin, subfamily a, member 4 | GO:0019827 | stem cell maintenance |
| 208794_s_at | 6597 | SMARCA4 | SWI/SNF related, matrix associated, actin dependent regulator of chromatin, subfamily a, member 4 | GO:0019827 | stem cell maintenance |
| 208991_at | 6774 | STAT3 | signal transducer and activator of transcription 3 (acute-phase response factor) | GO:0035019 | somatic stem cell maintenance |
| 208992_s_at | 6774 | STAT3 | signal transducer and activator of transcription 3 (acute-phase response factor) | GO:0035019 | somatic stem cell maintenance |
| 208996_s_at | 5432 | POLR2C | polymerase (RNA) II (DNA directed) polypeptide C, 33kDa | GO:0035019 | somatic stem cell maintenance |
| 209022_at | 10735 | STAG2 | stromal antigen 2 | GO:0019827 | stem cell maintenance |
| 209023_s_at | 10735 | STAG2 | stromal antigen 2 | GO:0019827 | stem cell maintenance |
| 209097_s_at | 182 | JAG1 | jagged 1 | GO:0097150 | neuronal stem cell maintenance |
| 209098_s_at | 182 | JAG1 | jagged 1 | GO:0097150 | neuronal stem cell maintenance |
| 209099_x_at | 182 | JAG1 | jagged 1 | GO:0097150 | neuronal stem cell maintenance |
| 209257_s_at | 9126 | SMC3 | structural maintenance of chromosomes 3 | GO:0019827 | stem cell maintenance |
| 209258_s_at | 9126 | SMC3 | structural maintenance of chromosomes 3 | GO:0019827 | stem cell maintenance |
| 209259_s_at | 9126 | SMC3 | structural maintenance of chromosomes 3 | GO:0019827 | stem cell maintenance |
| 209265_s_at | 56339 | METTL3 | methyltransferase like 3 | GO:0019827 | stem cell maintenance |
| 209302_at | 5437 | POLR2H | polymerase (RNA) II (DNA directed) polypeptide H | GO:0035019 | somatic stem cell maintenance |
| 209362_at | 9412 | MED21 | mediator complex subunit 21 | GO:0019827 | stem cell maintenance |
| 209363_s_at | 9412 | MED21 | mediator complex subunit 21 | GO:0019827 | stem cell maintenance |
| 209454_s_at | 7005 | TEAD3 | TEA domain family member 3 | GO:1902459 | positive regulation of stem cell maintenance |
| 209468_at | 4041 | LRP5 | low density lipoprotein receptor-related protein 5 | GO:0035019 | somatic stem cell maintenance |
| 209511_at | 5435 | POLR2F | polymerase (RNA) II (DNA directed) polypeptide F | GO:0035019 | somatic stem cell maintenance |
| 209704_at | 22823 | MTF2 | metal response element binding transcription factor 2 | GO:0019827 | stem cell maintenance |
| 209705_at | 22823 | MTF2 | metal response element binding transcription factor 2 | GO:0019827 | stem cell maintenance |
| 209710_at | 2624 | GATA2 | GATA binding protein 2 | GO:0035019 | somatic stem cell maintenance |
| 209954_x_at | 6760 | SS18 | synovial sarcoma translocation, chromosome 18 | GO:0097150 | neuronal stem cell maintenance |
| 209995_s_at | 8115 | TCL1A | T-cell leukemia/lymphoma 1A | GO:0019827 | stem cell maintenance |
| 209996_x_at | 5108 | PCM1 | pericentriolar material 1 | GO:0097150 | neuronal stem cell maintenance |
| 209997_x_at | 5108 | PCM1 | pericentriolar material 1 | GO:0097150 | neuronal stem cell maintenance |
| 210248_at | 7476 | WNT7A | wingless-type MMTV integration site family, member 7A | GO:0035019 | somatic stem cell maintenance |
| 210358_x_at | 2624 | GATA2 | GATA binding protein 2 | GO:0035019 | somatic stem cell maintenance |
| 210655_s_at | 2309 | FOXO3 | forkhead box O3 | GO:0097150 | neuronal stem cell maintenance |
| 210756_s_at | 4853 | NOTCH2 | notch 2 | GO:0019827 | stem cell maintenance |
| 211141_s_at | 4849 | CNOT3 | CCR4-NOT transcription complex, subunit 3 | GO:2000036 | regulation of stem cell maintenance |
| 211342_x_at | 9968 | MED12 | mediator complex subunit 12 | GO:0019827 | stem cell maintenance |
| 211412_at | 23569 | PADI4 | peptidyl arginine deiminase, type IV | GO:0019827 | stem cell maintenance |
| 211413_s_at | 23569 | PADI4 | peptidyl arginine deiminase, type IV | GO:0019827 | stem cell maintenance |
| 211730_s_at | 5441 | POLR2L | polymerase (RNA) II (DNA directed) polypeptide L, 7.6kDa | GO:0035019 | somatic stem cell maintenance |
| 211808_s_at | 1387 | CREBBP | CREB binding protein | GO:0030718 | germ-line stem cell maintenance |
| 211974_x_at | 3516 | RBPJ | recombination signal binding protein for immunoglobulin kappa J region | GO:0035019 | somatic stem cell maintenance |
| 212148_at | 5087 | PBX1 | pre-B-cell leukemia homeobox 1 | GO:0035019 | somatic stem cell maintenance |
| 212151_at | 5087 | PBX1 | pre-B-cell leukemia homeobox 1 | GO:0035019 | somatic stem cell maintenance |
| 212301_at | 23168 | RTF1 | Rtf1, Paf1/RNA polymerase II complex component, homolog (S. cerevisiae) | GO:0019827 | stem cell maintenance |
| 212302_at | 23168 | RTF1 | Rtf1, Paf1/RNA polymerase II complex component, homolog (S. cerevisiae) | GO:0019827 | stem cell maintenance |
| 212377_s_at | 4853 | NOTCH2 | notch 2 | GO:0019827 | stem cell maintenance |
| 212469_at | 25836 | NIPBL | Nipped-B homolog (Drosophila) | GO:0019827 | stem cell maintenance |
| 212483_at | 25836 | NIPBL | Nipped-B homolog (Drosophila) | GO:0019827 | stem cell maintenance |
| 212520_s_at | 6597 | SMARCA4 | SWI/SNF related, matrix associated, actin dependent regulator of chromatin, subfamily a, member 4 | GO:0019827 | stem cell maintenance |
| 212704_at | 23318 | ZCCHC11 | zinc finger, CCHC domain containing 11 | GO:0019827 | stem cell maintenance |
| 212759_s_at | 6934 | TCF7L2 | transcription factor 7-like 2 (T-cell specific, HMG-box) | GO:0035019 | somatic stem cell maintenance |
| 212761_at | 6934 | TCF7L2 | transcription factor 7-like 2 (T-cell specific, HMG-box) | GO:0035019 | somatic stem cell maintenance |
| 212762_s_at | 6934 | TCF7L2 | transcription factor 7-like 2 (T-cell specific, HMG-box) | GO:0035019 | somatic stem cell maintenance |
| 212782_x_at | 5439 | POLR2J | polymerase (RNA) II (DNA directed) polypeptide J, 13.3kDa | GO:0035019 | somatic stem cell maintenance |
| 212888_at | 23405 | DICER1 | dicer 1, ribonuclease type III | GO:0019827 | stem cell maintenance |
| 212955_s_at | 5438 | POLR2I | polymerase (RNA) II (DNA directed) polypeptide I, 14.5kDa | GO:0035019 | somatic stem cell maintenance |
| 213043_s_at | 9862 | MED24 | mediator complex subunit 24 | GO:0019827 | stem cell maintenance |
| 213171_s_at | 10893 | MMP24 | matrix metallopeptidase 24 (membrane-inserted) | GO:0097150 | neuronal stem cell maintenance |
| 213202_at | 9739 | SETD1A | SET domain containing 1A | GO:0019827 | stem cell maintenance |
| 213229_at | 23405 | DICER1 | dicer 1, ribonuclease type III | GO:0019827 | stem cell maintenance |
| 213342_at | 10413 | YAP1 | Yes-associated protein 1 | GO:0035019 | somatic stem cell maintenance |
| 213578_at | 657 | BMPR1A | bone morphogenetic protein receptor, type IA | GO:0019827 | stem cell maintenance |
| 213653_at | 56339 | METTL3 | methyltransferase like 3 | GO:0019827 | stem cell maintenance |
| 213665_at | 6659 | SOX4 | SRY (sex determining region Y)-box 4 | GO:0035019 | somatic stem cell maintenance |
| 213668_s_at | 6659 | SOX4 | SRY (sex determining region Y)-box 4 | GO:0035019 | somatic stem cell maintenance |
| 213719_s_at | 6597 | SMARCA4 | SWI/SNF related, matrix associated, actin dependent regulator of chromatin, subfamily a, member 4 | GO:0019827 | stem cell maintenance |
| 213720_s_at | 6597 | SMARCA4 | SWI/SNF related, matrix associated, actin dependent regulator of chromatin, subfamily a, member 4 | GO:0019827 | stem cell maintenance |
| 213721_at | 6657 | SOX2 | SRY (sex determining region Y)-box 2 | GO:0035019 | somatic stem cell maintenance |
| 213722_at | 6657 | SOX2 | SRY (sex determining region Y)-box 2 | GO:0035019 | somatic stem cell maintenance |
| 213755_s_at | 6497 | SKI | SKI proto-oncogene | GO:0035019 | somatic stem cell maintenance |
| 213887_s_at | 5434 | POLR2E | polymerase (RNA) II (DNA directed) polypeptide E, 25kDa | GO:0035019 | somatic stem cell maintenance |
| 213918_s_at | 25836 | NIPBL | Nipped-B homolog (Drosophila) | GO:0019827 | stem cell maintenance |
| 214118_x_at | 5108 | PCM1 | pericentriolar material 1 | GO:0097150 | neuronal stem cell maintenance |
| 214127_s_at | 51593 | SRRT | serrate, RNA effector molecule | GO:0097150 | neuronal stem cell maintenance |
| 214144_at | 5433 | POLR2D | polymerase (RNA) II (DNA directed) polypeptide D | GO:0035019 | somatic stem cell maintenance |
| 214178_s_at | 6657 | SOX2 | SRY (sex determining region Y)-box 2 | GO:0035019 | somatic stem cell maintenance |
| 214263_x_at | 5432 | POLR2C | polymerase (RNA) II (DNA directed) polypeptide C, 33kDa | GO:0035019 | somatic stem cell maintenance |
| 214275_at | 9968 | MED12 | mediator complex subunit 12 | GO:0019827 | stem cell maintenance |
| 214360_at | 6597 | SMARCA4 | SWI/SNF related, matrix associated, actin dependent regulator of chromatin, subfamily a, member 4 | GO:0019827 | stem cell maintenance |
| 214700_x_at | 55183 | RIF1 | replication timing regulatory factor 1 | GO:0019827 | stem cell maintenance |
| 214728_x_at | 6597 | SMARCA4 | SWI/SNF related, matrix associated, actin dependent regulator of chromatin, subfamily a, member 4 | GO:0019827 | stem cell maintenance |
| 214740_at | 5439 | POLR2J | polymerase (RNA) II (DNA directed) polypeptide J, 13.3kDa | GO:0035019 | somatic stem cell maintenance |
| 214872_at | 55183 | RIF1 | replication timing regulatory factor 1 | GO:0019827 | stem cell maintenance |
| 214937_x_at | 5108 | PCM1 | pericentriolar material 1 | GO:0097150 | neuronal stem cell maintenance |
| 215167_at | 9282 | MED14 | mediator complex subunit 14 | GO:0019827 | stem cell maintenance |
| 215310_at | 324 | APC | adenomatous polyposis coli | GO:0035019 | somatic stem cell maintenance |
| 215337_at | 9862 | MED24 | mediator complex subunit 24 | GO:0019827 | stem cell maintenance |
| 215371_at | 9442 | MED27 | mediator complex subunit 27 | GO:0019827 | stem cell maintenance |
| 215714_s_at | 6597 | SMARCA4 | SWI/SNF related, matrix associated, actin dependent regulator of chromatin, subfamily a, member 4 | GO:0019827 | stem cell maintenance |
| 215720_s_at | 4800 | NFYA | nuclear transcription factor Y, alpha | GO:2000036 | regulation of stem cell maintenance |
| 215804_at | 2041 | EPHA1 | EPH receptor A1 | GO:0035019 | somatic stem cell maintenance |
| 216035_x_at | 6934 | TCF7L2 | transcription factor 7-like 2 (T-cell specific, HMG-box) | GO:0035019 | somatic stem cell maintenance |
| 216037_x_at | 6934 | TCF7L2 | transcription factor 7-like 2 (T-cell specific, HMG-box) | GO:0035019 | somatic stem cell maintenance |
| 216071_x_at | 9968 | MED12 | mediator complex subunit 12 | GO:0019827 | stem cell maintenance |
| 216242_x_at | 5439 | POLR2J | polymerase (RNA) II (DNA directed) polypeptide J, 13.3kDa | GO:0035019 | somatic stem cell maintenance |
| 216260_at | 23405 | DICER1 | dicer 1, ribonuclease type III | GO:0019827 | stem cell maintenance |
| 216268_s_at | 182 | JAG1 | jagged 1 | GO:0097150 | neuronal stem cell maintenance |
| 216280_s_at | 23405 | DICER1 | dicer 1, ribonuclease type III | GO:0019827 | stem cell maintenance |
| 216281_at | 23405 | DICER1 | dicer 1, ribonuclease type III | GO:0019827 | stem cell maintenance |
| 216282_x_at | 5432 | POLR2C | polymerase (RNA) II (DNA directed) polypeptide C, 33kDa | GO:0035019 | somatic stem cell maintenance |
| 216361_s_at | 7994 | KAT6A | K(lysine) acetyltransferase 6A | GO:0035019 | somatic stem cell maintenance |
| 216511_s_at | 6934 | TCF7L2 | transcription factor 7-like 2 (T-cell specific, HMG-box) | GO:0035019 | somatic stem cell maintenance |
| 216684_s_at | 6760 | SS18 | synovial sarcoma translocation, chromosome 18 | GO:0097150 | neuronal stem cell maintenance |
| 216925_s_at | 6886 | TAL1 | T-cell acute lymphocytic leukemia 1 | GO:2000036 | regulation of stem cell maintenance |
| 216928_at | 6886 | TAL1 | T-cell acute lymphocytic leukemia 1 | GO:2000036 | regulation of stem cell maintenance |
| 216933_x_at | 324 | APC | adenomatous polyposis coli | GO:0035019 | somatic stem cell maintenance |
| 217120_s_at | 9282 | MED14 | mediator complex subunit 14 | GO:0019827 | stem cell maintenance |
| 217399_s_at | 2309 | FOXO3 | forkhead box O3 | GO:0097150 | neuronal stem cell maintenance |
| 217415_at | 5430 | POLR2A | polymerase (RNA) II (DNA directed) polypeptide A, 220kDa | GO:0035019 | somatic stem cell maintenance |
| 217420_s_at | 5430 | POLR2A | polymerase (RNA) II (DNA directed) polypeptide A, 220kDa | GO:0035019 | somatic stem cell maintenance |
| 217421_at | 55124 | PIWIL2 | piwi-like RNA-mediated gene silencing 2 | GO:0030718 | germ-line stem cell maintenance |
| 217555_at | 8243 | SMC1A | structural maintenance of chromosomes 1A | GO:0019827 | stem cell maintenance |
| 217594_at | 23318 | ZCCHC11 | zinc finger, CCHC domain containing 11 | GO:0019827 | stem cell maintenance |
| 217798_at | 4848 | CNOT2 | CCR4-NOT transcription complex, subunit 2 | GO:2000036 | regulation of stem cell maintenance |
| 217854_s_at | 5434 | POLR2E | polymerase (RNA) II (DNA directed) polypeptide E, 25kDa | GO:0035019 | somatic stem cell maintenance |
| 218276_s_at | 60485 | SAV1 | salvador family WW domain containing protein 1 | GO:2000036 | regulation of stem cell maintenance |
| 218438_s_at | 80306 | MED28 | mediator complex subunit 28 | GO:0019827 | stem cell maintenance |
| 218578_at | 79577 | CDC73 | cell division cycle 73 | GO:0019827 | stem cell maintenance |
| 218629_at | 6608 | SMO | smoothened, frizzled class receptor | GO:2000036 | regulation of stem cell maintenance |
| 218902_at | 4851 | NOTCH1 | notch 1 | GO:0097150 | neuronal stem cell maintenance |
| 219108_x_at | 6760 | SS18 | synovial sarcoma translocation, chromosome 18 | GO:0097150 | neuronal stem cell maintenance |
| 219379_x_at | 140467 | ZNF358 | zinc finger protein 358 | GO:0019827 | stem cell maintenance |
| 219682_s_at | 6926 | TBX3 | T-box 3 | GO:0019827 | stem cell maintenance |
| 219823_at | 79727 | LIN28A | lin-28 homolog A (C. elegans) | GO:0019827 | stem cell maintenance |
| 219918_s_at | 259266 | ASPM | asp (abnormal spindle) homolog, microcephaly associated (Drosophila) | GO:0097150 | neuronal stem cell maintenance |
| 220001_at | 23569 | PADI4 | peptidyl arginine deiminase, type IV | GO:0019827 | stem cell maintenance |
| 220184_at | 79923 | NANOG | Nanog homeobox | GO:0019827 | stem cell maintenance |
| 220266_s_at | 9314 | KLF4 | Kruppel-like factor 4 (gut) | GO:0019827 | stem cell maintenance |
| 220686_s_at | 55124 | PIWIL2 | piwi-like RNA-mediated gene silencing 2 | GO:0030718 | germ-line stem cell maintenance |
| 220689_at | 4838 | NODAL | nodal growth differentiation factor | GO:0019827 | stem cell maintenance |
| 220714_at | 63978 | PRDM14 | PR domain containing 14 | GO:0030718 | germ-line stem cell maintenance |
| 220928_s_at | 63976 | PRDM16 | PR domain containing 16 | GO:0035019 | somatic stem cell maintenance |
| 221016_s_at | 83439 | TCF7L1 | transcription factor 7-like 1 (T-cell specific, HMG-box) | GO:0035019 | somatic stem cell maintenance |
| 221198_at | 6343 | SCT | secretin | GO:0097150 | neuronal stem cell maintenance |
| 221517_s_at | 9440 | MED17 | mediator complex subunit 17 | GO:0019827 | stem cell maintenance |
| 221598_s_at | 9442 | MED27 | mediator complex subunit 27 | GO:0019827 | stem cell maintenance |
| 221841_s_at | 9314 | KLF4 | Kruppel-like factor 4 (gut) | GO:0019827 | stem cell maintenance |
| 222046_at | 51593 | SRRT | serrate, RNA effector molecule | GO:0097150 | neuronal stem cell maintenance |
| 222047_s_at | 51593 | SRRT | serrate, RNA effector molecule | GO:0097150 | neuronal stem cell maintenance |
| 222175_s_at | 51586 | MED15 | mediator complex subunit 15 | GO:0019827 | stem cell maintenance |
| 222181_at | 4848 | CNOT2 | CCR4-NOT transcription complex, subunit 2 | GO:2000036 | regulation of stem cell maintenance |
| 222182_s_at | 4848 | CNOT2 | CCR4-NOT transcription complex, subunit 2 | GO:2000036 | regulation of stem cell maintenance |
| 222573_s_at | 60485 | SAV1 | salvador family WW domain containing protein 1 | GO:2000036 | regulation of stem cell maintenance |
| 222635_s_at | 80306 | MED28 | mediator complex subunit 28 | GO:0019827 | stem cell maintenance |
| 222636_at | 80306 | MED28 | mediator complex subunit 28 | GO:0019827 | stem cell maintenance |
| 222917_s_at | 6926 | TBX3 | T-box 3 | GO:0019827 | stem cell maintenance |
| 223115_at | 9440 | MED17 | mediator complex subunit 17 | GO:0019827 | stem cell maintenance |
| 223247_at | 84246 | MED10 | mediator complex subunit 10 | GO:0019827 | stem cell maintenance |
| 223439_at | 79576 | NKAP | NFKB activating protein | GO:0019827 | stem cell maintenance |
| 223508_at | 4851 | NOTCH1 | notch 1 | GO:0097150 | neuronal stem cell maintenance |
| 223679_at | 1499 | CTNNB1 | catenin (cadherin-associated protein), beta 1, 88kDa | GO:0019827 | stem cell maintenance |
| 223762_at | 6944 | VPS72 | vacuolar protein sorting 72 homolog (S. cerevisiae) | GO:0035019 | somatic stem cell maintenance |
| 223858_at | 2103 | ESRRB | estrogen-related receptor beta | GO:0019827 | stem cell maintenance |
| 223976_at | 84750 | FUT10 | fucosyltransferase 10 (alpha (1,3) fucosyltransferase) | GO:0097150 | neuronal stem cell maintenance |
| 224215_s_at | 28514 | DLL1 | delta-like 1 (Drosophila) | GO:0097150 | neuronal stem cell maintenance |
| 224359_s_at | 84376 | HOOK3 | hook microtubule-tethering protein 3 | GO:0097150 | neuronal stem cell maintenance |
| 224416_s_at | 80306 | MED28 | mediator complex subunit 28 | GO:0019827 | stem cell maintenance |
| 224889_at | 2309 | FOXO3 | forkhead box O3 | GO:0097150 | neuronal stem cell maintenance |
| 224891_at | 2309 | FOXO3 | forkhead box O3 | GO:0097150 | neuronal stem cell maintenance |
| 224894_at | 10413 | YAP1 | Yes-associated protein 1 | GO:0035019 | somatic stem cell maintenance |
| 224895_at | 10413 | YAP1 | Yes-associated protein 1 | GO:0035019 | somatic stem cell maintenance |
| 225289_at | 6774 | STAT3 | signal transducer and activator of transcription 3 (acute-phase response factor) | GO:0035019 | somatic stem cell maintenance |
| 225544_at | 6926 | TBX3 | T-box 3 | GO:0019827 | stem cell maintenance |
| 226029_at | 57216 | VANGL2 | VANGL planar cell polarity protein 2 | GO:0035019 | somatic stem cell maintenance |
| 226260_x_at | 140467 | ZNF358 | zinc finger protein 358 | GO:0019827 | stem cell maintenance |
| 226391_at | 673 | BRAF | B-Raf proto-oncogene, serine/threonine kinase | GO:0035019 | somatic stem cell maintenance |
| 226395_at | 84376 | HOOK3 | hook microtubule-tethering protein 3 | GO:0097150 | neuronal stem cell maintenance |
| 226503_at | 55183 | RIF1 | replication timing regulatory factor 1 | GO:0019827 | stem cell maintenance |
| 226547_at | 7994 | KAT6A | K(lysine) acetyltransferase 6A | GO:0035019 | somatic stem cell maintenance |
| 226563_at | 4087 | SMAD2 | SMAD family member 2 | GO:0035019 | somatic stem cell maintenance |
| 226821_at | 55183 | RIF1 | replication timing regulatory factor 1 | GO:0019827 | stem cell maintenance |
| 227067_x_at | 4853 | NOTCH2 | notch 2 | GO:0019827 | stem cell maintenance |
| 227273_at | 657 | BMPR1A | bone morphogenetic protein receptor, type IA | GO:0019827 | stem cell maintenance |
| 227504_s_at | 80306 | MED28 | mediator complex subunit 28 | GO:0019827 | stem cell maintenance |
| 227505_at | 80306 | MED28 | mediator complex subunit 28 | GO:0019827 | stem cell maintenance |
| 227597_at | 129563 | DIS3L2 | DIS3 like 3'-5' exoribonuclease 2 | GO:0019827 | stem cell maintenance |
| 227601_at | 57721 | METTL14 | methyltransferase like 14 | GO:0019827 | stem cell maintenance |
| 227616_at | 283149 | BCL9L | B-cell CLL/lymphoma 9-like | GO:0035019 | somatic stem cell maintenance |
| 227681_at | 678 | ZFP36L2 | ZFP36 ring finger protein-like 2 | GO:0035019 | somatic stem cell maintenance |
| 227746_at | 1994 | ELAVL1 | ELAV like RNA binding protein 1 | GO:2000036 | regulation of stem cell maintenance |
| 227757_at | 8451 | CUL4A | cullin 4A | GO:0035019 | somatic stem cell maintenance |
| 227766_at | 3981 | LIG4 | ligase IV, DNA, ATP-dependent | GO:0035019 | somatic stem cell maintenance |
| 227786_at | 90390 | MED30 | mediator complex subunit 30 | GO:0019827 | stem cell maintenance |
| 227787_s_at | 90390 | MED30 | mediator complex subunit 30 | GO:0019827 | stem cell maintenance |
| 227938_s_at | 28514 | DLL1 | delta-like 1 (Drosophila) | GO:0097150 | neuronal stem cell maintenance |
| 228038_at | 6657 | SOX2 | SRY (sex determining region Y)-box 2 | GO:0035019 | somatic stem cell maintenance |
| 228065_at | 283149 | BCL9L | B-cell CLL/lymphoma 9-like | GO:0035019 | somatic stem cell maintenance |
| 228177_at | 1387 | CREBBP | CREB binding protein | GO:0030718 | germ-line stem cell maintenance |
| 228344_s_at | 6926 | TBX3 | T-box 3 | GO:0019827 | stem cell maintenance |
| 228413_s_at | 6422 | SFRP1 | secreted frizzled-related protein 1 | GO:0035019 | somatic stem cell maintenance |
| 228431_at | 4800 | NFYA | nuclear transcription factor Y, alpha | GO:2000036 | regulation of stem cell maintenance |
| 228433_at | 4800 | NFYA | nuclear transcription factor Y, alpha | GO:2000036 | regulation of stem cell maintenance |
| 228484_s_at | 2308 | FOXO1 | forkhead box O1 | GO:0097150 | neuronal stem cell maintenance |
| 228656_at | 5629 | PROX1 | prospero homeobox 1 | GO:0097150 | neuronal stem cell maintenance |
| 228905_at | 5108 | PCM1 | pericentriolar material 1 | GO:0097150 | neuronal stem cell maintenance |
| 228906_at | 80312 | TET1 | tet methylcytosine dioxygenase 1 | GO:0019827 | stem cell maintenance |
| 228992_at | 80306 | MED28 | mediator complex subunit 28 | GO:0019827 | stem cell maintenance |
| 229143_at | 4849 | CNOT3 | CCR4-NOT transcription complex, subunit 3 | GO:2000036 | regulation of stem cell maintenance |
| 229215_at | 430 | ASCL2 | achaete-scute family bHLH transcription factor 2 | GO:0035019 | somatic stem cell maintenance |
| 229265_at | 6497 | SKI | SKI proto-oncogene | GO:0035019 | somatic stem cell maintenance |
| 229376_at | 5629 | PROX1 | prospero homeobox 1 | GO:0097150 | neuronal stem cell maintenance |
| 229540_at | 3516 | RBPJ | recombination signal binding protein for immunoglobulin kappa J region | GO:0035019 | somatic stem cell maintenance |
| 229565_x_at | 6926 | TBX3 | T-box 3 | GO:0019827 | stem cell maintenance |
| 229576_s_at | 6926 | TBX3 | T-box 3 | GO:0019827 | stem cell maintenance |
| 229591_at | 4041 | LRP5 | low density lipoprotein receptor-related protein 5 | GO:0035019 | somatic stem cell maintenance |
| 229661_at | 57167 | SALL4 | spalt-like transcription factor 4 | GO:0035019 | somatic stem cell maintenance |
| 229828_at | 79577 | CDC73 | cell division cycle 73 | GO:0019827 | stem cell maintenance |
| 229924_s_at | 182 | JAG1 | jagged 1 | GO:0097150 | neuronal stem cell maintenance |
| 230555_s_at | 90390 | MED30 | mediator complex subunit 30 | GO:0019827 | stem cell maintenance |
| 230832_at | 23168 | RTF1 | Rtf1, Paf1/RNA polymerase II complex component, homolog (S. cerevisiae) | GO:0019827 | stem cell maintenance |
| 230916_at | 4838 | NODAL | nodal growth differentiation factor | GO:0019827 | stem cell maintenance |
| 230979_at | 657 | BMPR1A | bone morphogenetic protein receptor, type IA | GO:0019827 | stem cell maintenance |
| 231079_at | 79923 | NANOG | Nanog homeobox | GO:0019827 | stem cell maintenance |
| 231272_at | 9443 | MED7 | mediator complex subunit 7 | GO:0019827 | stem cell maintenance |
| 231548_at | 2309 | FOXO3 | forkhead box O3 | GO:0097150 | neuronal stem cell maintenance |
| 231762_at | 2255 | FGF10 | fibroblast growth factor 10 | GO:0035019 | somatic stem cell maintenance |
| 231776_at | 8320 | EOMES | eomesodermin | GO:0019827 | stem cell maintenance |
| 231798_at | 9241 | NOG | noggin | GO:0035019 | somatic stem cell maintenance |
| 232238_at | 259266 | ASPM | asp (abnormal spindle) homolog, microcephaly associated (Drosophila) | GO:0097150 | neuronal stem cell maintenance |
| 232424_at | 63976 | PRDM16 | PR domain containing 16 | GO:0035019 | somatic stem cell maintenance |
| 232466_at | 8451 | CUL4A | cullin 4A | GO:0035019 | somatic stem cell maintenance |
| 232483_at | 9440 | MED17 | mediator complex subunit 17 | GO:0019827 | stem cell maintenance |
| 232643_at | 5435 | POLR2F | polymerase (RNA) II (DNA directed) polypeptide F | GO:0035019 | somatic stem cell maintenance |
| 233054_at | 4848 | CNOT2 | CCR4-NOT transcription complex, subunit 2 | GO:2000036 | regulation of stem cell maintenance |
| 233780_at | 55183 | RIF1 | replication timing regulatory factor 1 | GO:0019827 | stem cell maintenance |
| 233781_s_at | 55183 | RIF1 | replication timing regulatory factor 1 | GO:0019827 | stem cell maintenance |
| 233802_at | 80306 | MED28 | mediator complex subunit 28 | GO:0019827 | stem cell maintenance |
| 234491_s_at | 60485 | SAV1 | salvador family WW domain containing protein 1 | GO:2000036 | regulation of stem cell maintenance |
| 235096_at | 123169 | LEO1 | Leo1, Paf1/RNA polymerase II complex component, homolog (S. cerevisiae) | GO:0019827 | stem cell maintenance |
| 235105_at | 80306 | MED28 | mediator complex subunit 28 | GO:0019827 | stem cell maintenance |
| 235114_x_at | 84376 | HOOK3 | hook microtubule-tethering protein 3 | GO:0097150 | neuronal stem cell maintenance |
| 235196_at | 79577 | CDC73 | cell division cycle 73 | GO:0019827 | stem cell maintenance |
| 235472_at | 84750 | FUT10 | fucosyltransferase 10 (alpha (1,3) fucosyltransferase) | GO:0097150 | neuronal stem cell maintenance |
| 235552_at | 57721 | METTL14 | methyltransferase like 14 | GO:0019827 | stem cell maintenance |
| 235598_at | 4087 | SMAD2 | SMAD family member 2 | GO:0035019 | somatic stem cell maintenance |
| 235725_at | 4089 | SMAD4 | SMAD family member 4 | GO:0035019 | somatic stem cell maintenance |
| 235858_at | 1387 | CREBBP | CREB binding protein | GO:0030718 | germ-line stem cell maintenance |
| 236060_at | 57721 | METTL14 | methyltransferase like 14 | GO:0019827 | stem cell maintenance |
| 236094_at | 6934 | TCF7L2 | transcription factor 7-like 2 (T-cell specific, HMG-box) | GO:0035019 | somatic stem cell maintenance |
| 236192_at | 84376 | HOOK3 | hook microtubule-tethering protein 3 | GO:0097150 | neuronal stem cell maintenance |
| 236402_at | 673 | BRAF | B-Raf proto-oncogene, serine/threonine kinase | GO:0035019 | somatic stem cell maintenance |
| 236620_at | 55183 | RIF1 | replication timing regulatory factor 1 | GO:0019827 | stem cell maintenance |
| 237896_at | 4838 | NODAL | nodal growth differentiation factor | GO:0019827 | stem cell maintenance |
| 238602_at | 129563 | DIS3L2 | DIS3 like 3'-5' exoribonuclease 2 | GO:0019827 | stem cell maintenance |
| 238729_x_at | 60485 | SAV1 | salvador family WW domain containing protein 1 | GO:2000036 | regulation of stem cell maintenance |
| 239002_at | 259266 | ASPM | asp (abnormal spindle) homolog, microcephaly associated (Drosophila) | GO:0097150 | neuronal stem cell maintenance |
| 239020_at | 84750 | FUT10 | fucosyltransferase 10 (alpha (1,3) fucosyltransferase) | GO:0097150 | neuronal stem cell maintenance |
| 239230_at | 388585 | HES5 | hes family bHLH transcription factor 5 | GO:0097150 | neuronal stem cell maintenance |
| 239271_at | 4087 | SMAD2 | SMAD family member 2 | GO:0035019 | somatic stem cell maintenance |
| 239303_at | 55124 | PIWIL2 | piwi-like RNA-mediated gene silencing 2 | GO:0030718 | germ-line stem cell maintenance |
| 239688_at | 8243 | SMC1A | structural maintenance of chromosomes 1A | GO:0019827 | stem cell maintenance |
| 240098_at | 55183 | RIF1 | replication timing regulatory factor 1 | GO:0019827 | stem cell maintenance |
| 240301_at | 151871 | DPPA2 | developmental pluripotency associated 2 | GO:0019827 | stem cell maintenance |
| 240421_x_at | 60485 | SAV1 | salvador family WW domain containing protein 1 | GO:2000036 | regulation of stem cell maintenance |
| 241055_at | 2034 | EPAS1 | endothelial PAS domain protein 1 | GO:0035019 | somatic stem cell maintenance |
| 241482_at | 2041 | EPHA1 | EPH receptor A1 | GO:0035019 | somatic stem cell maintenance |
| 241609_at | 27022 | FOXD3 | forkhead box D3 | GO:0035019 | somatic stem cell maintenance |
| 241612_at | 27022 | FOXD3 | forkhead box D3 | GO:0035019 | somatic stem cell maintenance |
| 241689_at | 57721 | METTL14 | methyltransferase like 14 | GO:0019827 | stem cell maintenance |
| 241820_at | 55183 | RIF1 | replication timing regulatory factor 1 | GO:0019827 | stem cell maintenance |
| 242111_at | 56339 | METTL3 | methyltransferase like 3 | GO:0019827 | stem cell maintenance |
| 242119_at | 5629 | PROX1 | prospero homeobox 1 | GO:0097150 | neuronal stem cell maintenance |
| 242352_at | 25836 | NIPBL | Nipped-B homolog (Drosophila) | GO:0019827 | stem cell maintenance |
| 242360_at | 9079 | LDB2 | LIM domain binding 2 | GO:0035019 | somatic stem cell maintenance |
| 242539_at | 129563 | DIS3L2 | DIS3 like 3'-5' exoribonuclease 2 | GO:0019827 | stem cell maintenance |
| 242731_x_at | 657 | BMPR1A | bone morphogenetic protein receptor, type IA | GO:0019827 | stem cell maintenance |
| 243213_at | 6774 | STAT3 | signal transducer and activator of transcription 3 (acute-phase response factor) | GO:0035019 | somatic stem cell maintenance |
| 243234_at | 6926 | TBX3 | T-box 3 | GO:0019827 | stem cell maintenance |
| 243668_at | 80306 | MED28 | mediator complex subunit 28 | GO:0019827 | stem cell maintenance |
| 243829_at | 673 | BRAF | B-Raf proto-oncogene, serine/threonine kinase | GO:0035019 | somatic stem cell maintenance |
| 244304_at | 129563 | DIS3L2 | DIS3 like 3'-5' exoribonuclease 2 | GO:0019827 | stem cell maintenance |
| 244660_at | 1994 | ELAVL1 | ELAV like RNA binding protein 1 | GO:2000036 | regulation of stem cell maintenance |
| 35160_at | 8861 | LDB1 | LIM domain binding 1 | GO:0035019 | somatic stem cell maintenance |
| 39318_at | 8115 | TCL1A | T-cell leukemia/lymphoma 1A | GO:0019827 | stem cell maintenance |
| 49679_s_at | 10893 | MMP24 | matrix metallopeptidase 24 (membrane-inserted) | GO:0097150 | neuronal stem cell maintenance |
| 51176_at | 9442 | MED27 | mediator complex subunit 27 | GO:0019827 | stem cell maintenance |

Table S3. Composition of the chemical cocktail (6C+3GF) for iMSC generation.

| **Components** | **500 ml** | **Company** |
| --- | --- | --- |
| Knockout DMEM | 475 ml | Invitrogen |
| AlbuMAX I | 5 g | Invitrogen |
| N2 supplement | 5 ml | Invitrogen |
| Nonessential amino acids (NEAA) | 5 ml | Invitrogen |
| SB202190 (p38i) | 10 μM | LC Laboratories |
| SP600125 (JNKi) | 10 μM | LC Laboratories |
| Go6983 (PKCi) | 0.5 μM | TOCRIS |
| Y-27632 (ROCKi) | 5 μM | LC Laboratories |
| PD0325901 (ERK1/2i) | 1 μM | LC Laboratories |
| CHIR99021 (GSK3βi) | 3 μM | LC Laboratories |
| Recombinant human LIF | 20 ng/ml | Peprotech |
| Recombinant bFGF | 8 ng/ml | Peprotech |
| Recombinant TGF-β1 | 1 ng/ml | Peprotech |
